# Supplementary material for: Interventions to promote access to eye care for non-Indigenous, non-dominant ethnic groups in high-income countries: a scoping review protocol
Source: BMJ Open. 2020 Jun 3;10(6):e033775. doi: 10.1136/bmjopen-2019-033775 (PMC7279662; doi:10.1136/bmjopen-2019-033775)
Supplement: Supplementary data [file bmjopen-2019-033775supp001.pdf]

Appendix: Search strategy

MEDLINE (Ovid)

1. exp Ophthalmology/
2. Optometry/
3. ((eye$ or ocular or vision) adj2 (care or health or service$)).tw.
4. visual acuity.tw.
5. (ophthalm$ or optomet$).tw.
6. exp Eye Diseases/
7. (glaucoma$ or ocular hypertension or cataract$).tw.
8. ((diabet$ or proliferat$) adj3 retinopath$).tw.
9. (diabet$ adj3 (eye$ or vision or visual$ or sight$)).tw.
10. (retinopath$ adj3 (eye$ or vision or visual$ or sight$)).tw.
11. (dilated adj2 fundus).tw.
12. (retinal adj2 exam$).tw.
13. (myop$ or hyperop$ or hypermetrop$ or anisometrop$ or ammetrop$ or astigmati$ or presbyop$).tw.
14. (refractive adj1 error$).tw.
15. Eyeglasses/
16. (spectacle or spectacles or glasses).tw.
17. (eyeglasses or eye glasses).tw.
18. or/1-17
19. Ethnic Groups/
20. Minority Groups/
21. ((ethnic$ or racial$ or cultur$) adj3 (group$ or minorit$ or population$ or diverse$ or origin$)).tw.
22. ((ethnic$ or racial$ or cultur$) adj3 (inequalit$ or inequit$ or disparit$ or equit$ or disadvantage$ or depriv$)).tw.
23. ((population$ or communit$) adj3 (divers$ or disadvantage$ or depriv$)).tw.
24. non-Indigenous.tw.
25. (visible adj1 minorit$).tw.
26. Refugees/
27. "emigrants and immigrants"/
28. (migrant$ or immigrant$ or emigrant$ or refugee$ or expatriate$).tw.
29. asylum seeker$.tw.
30. Urban Population/
31. urban poor.tw.
32. Cultural Characteristics/
33. Cross-Cultural Comparison/
34. Cultural Diversity/
35. Cultural competency/
36. Cultural deprivation/
37. exp African Continental Ancestry Group/
38. exp Asian Continental Ancestry Group/
39. Continental Population Groups/
40. geography.tw.
41. (Afr$ adj2 American$).tw.
42. (Afr$ adj2 Caribbean$).tw.
43. (west adj2 (india$ or indies)).tw.
44. (American$ adj1 black).tw.
45. (Asian$ or Indian$ or Pakistan$ or Bangladesh$ or Bengal$).tw.
46. ((Asia$ or Pacific) adj4 American$).tw.
47. ((Asia$ or Pacific) adj4 Islander$).tw.
48. (Hispanic or Latino or Latin American$ or Puerto Ric$ or Mexican$).tw.
49. or/19-48
50. Program Evaluation/
51. program$.tw.
52. ((educat$ or behaviour$ or behavior$ or improv$) adj3 (intervention$ or activat$)).tw.
53. Delivery of Health Care/
54. Health Services Accessibility/
55. Patient Acceptance of Health Care/
56. Health Promotion/
57. (health adj2 (promotion$ or knowledge or belief$)).tw.
58. Health Education/
59. (educat$ adj2 (information or material or leaflet)).tw.
60. Health Knowledge, Attitudes, Practice/
61. Patient Education as Topic/
62. Persuasive Communication/
63. "Surveys and Questionnaires"/
64. Questionnaires/
65. Focus Groups/
66. Health Surveys/
67. Health Care Surveys/

68. Interviews as Topic/
69. (questionnaire$ or survey$).tw.
70. (focus adj3 group$).tw.
71. exp Reminder Systems/
72. remind$.tw.
73. Telephone/
74. telephone.tw.
75. phone call.tw.
76. Health Behavior/
77. Behavior Therapy/
78. (behavioral or behavior or behaviour or behavioural).tw.
79. (increas$ adj3 (attend$ or uptake)).tw.
80. (approachability or acceptability or availability or affordability or appropriateness).tw.
81. (ability adj2 (perceive or seek or reach or pay or engage)).tw.
82. exp Vision Tests/
83. Mass Screening/
84. (vision adj3 (test$ or screen$ or assess$)).tw.
85. (eye$ adj3 (test$ or screen$ or assess$)).tw.
86. ((target$ or tailor$) adj3 intervention$).tw.
87. (cultural$ adj3 (sensitiv$ or appropriate)).tw.
88. or/50-87
89. 18 and 49 and 88
90. exp developing countries/
91. 89 not 90
92. prevalence.ti.
93. (genetic or mutation$ or autosomal or variant$).ti.
94. optical coherence tomography.tw.
95. (Latin American and Caribbean Health Sciences).tw.
96. or/92-95
97. 91 not 96
98. exp case report/
99. (case$ adj3 (report$ or series)).tw.
100. 98 or 99
101. 97 not 100

Embase (Ovid)

1. ophthalmology/
2. optometry/
3. optometrist/
4. ((eye$ or ocular or vision) adj2 (care or health or service$)).tw.
5. visual acuity.tw.
6. (ophthalm$ or optomet$).tw.
7. exp eye disease/
8. (glaucoma$ or ocular hypertension or cataract$).tw.
9. ((diabet$ or proliferat$) adj3 retinopath$).tw.
10. (diabet$ adj3 (eye$ or vision or visual$ or sight$)).tw.
11. (retinopath$ adj3 (eye$ or vision or visual$ or sight$)).tw.
12. eye fundus/
13. (dilated adj2 fundus).tw.
14. (retinal adj2 exam$).tw.
15. (myop$ or hyperop$ or hypermetrop$ or anisometrop$ or ammetrop$ or astigmati$ or presbyop$).tw.
16. (refractive adj1 error$).tw.
17. spectacles/
18. (spectacle or spectacles or glasses).tw.
19. (eyeglasses or eye glasses).tw.
20. or/1-19
21. "ethnic or racial aspects"/
22. ethnic group/
23. minority group/
24. ((ethnic$ or racial$ or cultur$) adj3 (group$ or minorit$ or population$ or diverse$ or origin$)).tw.
25. ((ethnic$ or racial$ or cultur$) adj3 (inequalit$ or inequit$ or disparit$ or equit$ or disadvantage$ or depriv$)).tw.
26. ((population$ or communit$) adj3 (divers$ or disadvantage$ or depriv$)).tw.
27. non-Indigenous.tw.
28. (visible adj1 minorit$).tw.
29. refugee/
30. asylum seeker/
31. "emigrants and immigrants"/
32. (migrant$ or immigrant$ or emigrant$ or refugee$ or expatriate$).tw.
33. asylum seeker$.tw.
34. urban population/

35. urban poor.tw.
36. cultural factor/
37. cultural diversity/
38. cultural competence/
39. cultural deprivation/
40. ancestry group/
41. exp african american/
42. african caribbean/
43. black person/
44. negro/
45. "Caribbean (person)"/
46. asian continental ancestry group/
47. asian american/
48. population group/
49. hispanic/
50. geography.tw.
51. (Afr$ adj2 American$).tw.
52. (Afr$ adj2 Caribbean$).tw.
53. (west adj2 (india$ or indies)).tw.
54. (American$ adj1 black).tw.
55. (Asian$ or Indian$ or Pakistan$ or Bangladesh$ or Bengal$).tw.
56. ((Asia$ or Pacific) adj4 American$).tw.
57. ((Asia$ or Pacific) adj4 Islander$).tw.
58. (Hispanic or Latino or Latin American$ or Puerto Ric$ or Mexican$).tw.
59. or/21-58
60. program evaluation/
61. program$.tw.
62. ((educat$ or behaviour$ or behavior$ or improv$) adj3 (intervention$ or activat$)).tw.
63. health care delivery/
64. patient attitude/
65. health promotion/
66. (health adj2 (promotion$ or knowledge or belief$)).tw.
67. health education/
68. (educat$ adj2 (information or material or leaflet)).tw.
69. attitude to health/
70. health behavior/
71. patient education/
72. persuasive communication/
73. questionnaires/
74. information processing/
75. health survey/
76. health care survey/
77. exp interview/
78. (questionnaire$ or survey$).tw.
79. (focus adj3 group$).tw.
80. reminder system/
81. remind$.tw.
82. telephone/
83. telephone interview/
84. telephone.tw.
85. phone call.tw.
86. health behavior/
87. behavior therapy/
88. behavior change/
89. (behavioral or behavior or behaviour or behavioural).tw.
90. (increas$ adj3 (attend$ or uptake)).tw.
91. (approachability or acceptability or availability or affordability or appropriateness).tw.
92. (ability adj2 (perceive or seek or reach or pay or engage)).tw.
93. vision test/
94. mass screening/
95. (vision adj3 (test$ or screen$ or assess$)).tw.
96. (eye$ adj3 (test$ or screen$ or assess$)).tw.
97. ((target$ or tailor$) adj3 intervention$).tw.
98. (cultural$ adj3 (sensitiv$ or appropriate)).tw.
99. or/60-98
100. 20 and 59 and 99
101. exp developing country/
102. 100 not 101
103. prevalence.ti.
104. (genetic or mutation$ or autosomal or variant$).ti.
105. optical coherence tomography.tw.
106. (Latin American and Caribbean Health Sciences).tw.
107. or/103-106
108. 102 not 107
109. exp case report/
110. (case$ adj3 (report$ or series)).tw.
111. or/109-110
112. 108 not 111
113. limit 112 to conference abstract status
114. 112 not 113

Global Health (Ovid)

1. eyes/
2. eye diseases/
3. vision/
4. vision disorders/
5. ((eye$ or ocular or vision) adj2 (care or health or service$)).tw.
6. visual acuity.tw.
7. (ophthalm$ or optomet$).tw.
8. (glaucoma$ or ocular hypertension or cataract$).tw.
9. ((diabet$ or proliferat$) adj3 retinopath$).tw.
10. (diabet$ adj3 (eye$ or vision or visual$ or sight$)).tw.
11. (retinopath$ adj3 (eye$ or vision or visual$ or sight$)).tw.
12. (dilated adj2 fundus).tw.
13. (retinal adj2 exam$).tw.
14. (myop$ or hyperop$ or hypermetrop$ or anisometrop$ or ammetrop$ or astigmati$ or presbyop$).tw.
15. (refractive adj1 error$).tw.
16. (spectacle or spectacles or glasses).tw.
17. (eyeglasses or eye glasses).tw.
18. or/1-17
19. ethnic groups/
20. ethnicity/
21. minorities/
22. ((ethnic$ or racial$ or cultur$) adj3 (group$ or minorit$ or population$ or diverse$ or origin$)).tw.
23. ((ethnic$ or racial$ or cultur$) adj3 (inequalit$ or inequit$ or disparit$ or equit$ or disadvantage$ or depriv$)).tw.
24. ((population$ or communit$) adj3 (divers$ or disadvantage$ or depriv$)).tw.
25. non-Indigenous.tw.
26. (visible adj1 minorit$).tw.
27. refugees/
28. immigrants/
29. immigration/
30. (migrant$ or immigrant$ or emigrant$ or refugee$ or expatriate$).tw.
31. asylum seeker$.tw.
32. urban population/
33. urban areas/
34. urban poor.tw.
35. geography.tw.
36. african americans/ or african-caribbeans/ or american indians/ or asians/ or black people/ or japanese americans/ or mexican-americans/ or pacific islanders/
37. (Afr$ adj2 American$).tw.
38. (Afr$ adj2 Caribbean$).tw.
39. (west adj2 (india$ or indies)).tw.
40. (American$ adj1 black).tw.
41. (Asian$ or Indian$ or Pakistan$ or Bangladesh$ or Bengal$).tw.
42. ((Asia$ or Pacific) adj4 American$).tw.
43. or/19-42
44. program evaluation/
45. programs/
46. program$.tw.
47. ((educat$ or behaviour$ or behavior$ or improv$) adj3 (intervention$ or activat$)).tw.
48. (primary health care or health services or community health or health policy or health care or public health or health programmes).sh.
49. health promotion/
50. (health adj2 (promotion$ or knowledge or belief$)).tw.
51. health education/
52. patient education/
53. (educat$ adj2 (information or material or leaflet)).tw.
54. attitude/
55. communication/
56. questionnaires/
57. discussion groups/
58. surveys/
59. (questionnaire$ or survey$).tw.
60. (focus adj3 group$).tw.
61. remind$.tw.
62. telephone/
63. telephone.tw.
64. phone call.tw.

65. behaviour/
66. (behavioral or behavior or behaviour or behavioural).tw.
67. (increas$ adj3 (attend$ or uptake)).tw.
68. (approachability or acceptability or availability or affordability or appropriateness).tw.
69. (ability adj2 (perceive or seek or reach or pay or engage)).tw.
70. screening/
71. (vision adj3 (test$ or screen$ or assess$)).tw.
72. (eye$ adj3 (test$ or screen$ or assess$)).tw.
73. ((target$ or tailor$) adj3 intervention$).tw.
74. (cultural$ adj3 (sensitiv$ or appropriate)).tw.
75. culture/
76. or/44-75
77. 18 and 43 and 76
78. exp developing countries/
79. 77 not 78
80. prevalence.ti.
81. (genetic or mutation$ or autosomal or variant$).ti.
82. optical coherence tomography.tw.
83. (Latin American and Caribbean Health Sciences).tw.
84. or/80-83
85. 79 not 84
86. exp case report/
87. (case$ adj3 (report$ or series)).tw.
88. 86 or 87
89. 85 not 88
