# Supplementary material for: Interventions to promote access to eye care for non-Indigenous, non-dominant ethnic groups in high-income countries: a scoping review protocol
Source: BMJ Open. 2020 Jun 3;10(6):e033775. doi: 10.1136/bmjopen-2019-033775 (PMC7279662; doi:10.1136/bmjopen-2019-033775)
Supplement: Author's manuscript [file bmjopen-2019-033775.draft_revisions.pdf]

BMJ Open

BMJ Open is committed to open peer review. As part of this commitment we make the peer review history of every article we publish publicly available.

When an article is published we post the peer reviewers' comments and the authors' responses online. We also post the versions of the paper that were used during peer review. These are the versions that the peer review comments apply to.

The versions of the paper that follow are the versions that were submitted during the peer review process. They are not the versions of record or the final published versions. They should not be cited or distributed as the published version of this manuscript.

BMJ Open is an open access journal and the full, final, typeset and author-corrected version of record of the manuscript is available on our site with no access controls, subscription charges or pay-per-view fees (http://bmjopen.bmj.com).

If you have any questions on BMJ Open's open peer review process please email info.bmjopen@bmj.com

BMJ Open

Interventions to promote access to eye care for non-Indigenous, non-dominant ethnic groups in high-income countries: a scoping review protocol

Journal:	*BMJ Open*
Manuscript ID	bmjopen-2019-033775
Article Type:	Protocol
Date Submitted by the Author:	22-Aug-2019
Complete List of Authors:	Hamm, Lisa; The University of Auckland, School of Optometry and Vision Science<br>Black, Joanna; The University of Auckland, School of Optometry and Vision Science<br>Burn, Helen; Brighton and Sussex University Hospitals NHS Trust<br>Grey, Corina; University of Auckland, School of Population Health<br>Harwood, Matire; University of Auckland, School of Population Health<br>Peiris-John, Roshini; The University of Auckland, School of Population Health<br>Gordon, Iris; London School of Hygiene and Tropical Medicine Faculty of Infectious and Tropical Diseases, International Centre for Eye Health<br>Evans, Jennifer; London School of Hygiene and Tropical Medicine Faculty of Infectious and Tropical Diseases, International Centre for Eye Health<br>Ramke, Jacqueline; London School of Hygiene and Tropical Medicine Faculty of Infectious and Tropical Diseases, International Centre for Eye Health; The University of Auckland, School of Optometry and Vision Science
Keywords:	ethnic minority, ethnic disparity, immigrants, eye care, optometry

SCHOLARONE™
Manuscripts

I, the Submitting Author has the right to grant and does grant on behalf of all authors of the Work (as defined in the below author licence), an exclusive licence and/or a non-exclusive licence for contributions from authors who are: i) UK Crown employees; ii) where BMJ has agreed a CC-BY licence shall apply, and/or iii) in accordance with the terms applicable for US Federal Government officers or employees acting as part of their official duties; on a worldwide, perpetual, irrevocable, royalty-free basis to BMJ Publishing Group Ltd ("BMJ") its licensees and where the relevant Journal is co-owned by BMJ to the co-owners of the Journal, to publish the Work in this journal and any other BMJ products and to exploit all rights, as set out in our licence.

The Submitting Author accepts and understands that any supply made under these terms is made by BMJ to the Submitting Author unless you are acting as an employee on behalf of your employer or a postgraduate student of an affiliated institution which is paying any applicable article publishing charge ("APC") for Open Access articles. Where the Submitting Author wishes to make the Work available on an Open Access basis (and intends to pay the relevant APC), the terms of reuse of such Open Access shall be governed by a Creative Commons licence – details of these licences and which Creative Commons licence will apply to this Work are set out in our licence referred to above.

Other than as permitted in any relevant BMJ Author's Self Archiving Policies, I confirm this Work has not been accepted for publication elsewhere, is not being considered for publication elsewhere and does not duplicate material already published. I confirm all authors consent to publication of this Work and authorise the granting of this licence.

For peer review only

Interventions to promote access to eye care for non-Indigenous, non-dominant ethnic groups in high-income countries: a scoping review protocol

Lisa M **Hamm**[1]	l.hamm@auckland.ac.nz	0000-0003-2777-7146
Joanna **Black**[1]	j.black@auckland.ac.nz	0000-0002-5100-8796
Helen **Burn**[2]	hburn88@gmail.com	0000-0002-1469-8169
Corina **Grey**[3]	c.grey@auckland.ac.nz	0000-0003-1751-1934
Matire **Harwood**[3]	m.harwood@auckland.ac.nz	0000-0003-1240-5139
Roshini **Peiris-John**[3]	r.peiris-john@auckland.ac.nz	0000-0001-7812-2268
Iris **Gordon**[2]	Iris.Gordon@lshtm.ac.uk	0000-0001-8143-8132
Jennifer R **Evans**[2]	Jennifer.Evans@lshtm.ac.uk	0000-0002-6137-2030
Jacqueline **Ramke**[1,2,3]	jacqueline.ramke@lshtm..ac.uk	0000-0002-5764-1306

1. School of Optometry & Vision Science, Faculty of Medicine and Health Sciences, The University of Auckland, Auckland, New Zealand
2. International Centre for Eye Health, London School of Hygiene and Tropical Medicine, London, United Kingdom
3. School of Population Health, Faculty of Medicine and Health Sciences, University of Auckland, Auckland, New Zealand

Article Summary

Author Contributors: JR conceived the idea for the review. LMH and JR drafted and revised the protocol with suggestions from JB, HB, CG, MH, RPJ and JE who reviewed the protocol and provided feedback on the draft. IG constructed the search.

Corresponding Author: Jacqueline Ramke (j.ramke@auckland.ac.nz)

Funding Statement: This work was supported by The University of Auckland Faculty Research Development Fund grant number 3716758.

Competing Interests: None declared. **Table Number**: 0. **Figure Number**: 0

Patient and Public Involvement Statement: As this was a review of existing literature, this research was done without specific patient involvement.

Data Sharing Statement: Data generated from this review will be available upon reasonable request from Jacqueline Ramke (j.ramke@auckland.ac.nz)

Keywords: non-dominant ethnic group, ethnic minority, ethnic disparity, immigrants, eye care, optometry, ophthalmology, access, service delivery, health

Word count: 2156

Strengths and limitations of this study

- This study will provide a comprehensive overview of interventions to improve access to eye care for non-Indigenous, non-dominant ethnic groups in high-income countries.
- The review will be comprehensive, including published literature of all study designs, without time period or language restrictions.
- A potential limitation could be a paucity of information on the topic.
- Another potential limitation is that the population of interest is difficult to define.

For peer review only

ABSTRACT

Introduction

For many people, settling in a new country is associated with a new identity as an ‘ethnic minority’, one that can remain through future generations. People who are culturally distinct from the dominant population group may experience a variety of barriers to accessing health care, including linguistic and cultural barriers in communication, navigation of an unfamiliar health system, and unconscious or overt discrimination. Here we outline the protocol of a scoping review to identify, describe and summarise interventions aimed at improving access to eye care for non-Indigenous, non-dominant ethnic groups residing in high-income countries.

Methods and analysis

We will search MEDLINE, Embase and Global Health from their inception with no date limits. We will include studies of any design that describe an intervention to promote access to eye care for non-Indigenous, non-dominant ethnic groups. Two authors will independently review titles, abstracts, and full-text articles for inclusion. Reference lists from all included articles will also be searched. In cases of disagreement between initial reviewers, a third author will help resolve the conflict. For each included article we will extract data about the target population, details of the intervention delivered and the effectiveness of or feedback from the intervention. Overall findings will be summarised with descriptive statistics and thematic analysis.

Ethics and dissemination

This review will summarise existing literature and as such ethics approval is not required. The scoping review is part of a larger project to improve access to eye care services for Māori and Pacific Peoples in Aotearoa New Zealand. We will publish the review in a peer-reviewed journal, and draft appropriate summaries for dissemination to the wider community. This wider community could include clinicians, policymakers, health service managers and organisations that work with vulnerable ethnic minorities.

For peer review only

For peer review only - http://bmjopen.bmj.com/site/about/guidelines.xhtml

INTRODUCTION

Rationale

Equitable access to health care is critically important, but it is a challenge to both define and achieve[1]. Health systems are often implicitly structured to meet the needs and preferences of members of the dominant group in any given population, which makes these systems more challenging for people with diverse backgrounds to navigate[2 3]. The axes of diversity vary widely (including socio-economic status, gender, sexual orientation, Indigeneity) and are often intersectional. Challenges in navigation of health care systems are compounded for people with a non-dominant ethnic background, because the health care seeker is more likely to look, speak, and communicate differently to their health care providers.[4]

The history of each ethnic group in a given place can influence how and to what extent health services strive to mitigate the vulnerabilities experienced by the group. For example, there is increasing recognition of overt institutional racism against colonised Indigenous populations and the impact this has on health care and health outcomes.[5] Formal efforts at restitution[6] have attempted to improve access to health care such as government-funded services to rural and remote areas with high Indigenous populations, and health facilities within Indigenous communities. For this reason, we are investigating service delivery models to improve access to eye care for Indigenous populations in a complementary scoping review,[7] and in the scoping review outlined here, we consider interventions to promote access to eye care for *non-Indigenous*, non-dominant ethnic groups.

A 'migrant' is a person who is living or has lived in a different place than they were born.[3] Using this definition, it was estimated that 3.4% of the global population (258 million people) were migrants in 2017.[8] People move away from their country of birth for a variety of reasons; many move for employment, others are forced from their home country because of civil unrest or violence, and others are moved through human trafficking and modern slavery.[3] Some migrants arrive in countries with a similar culture and language to their own, while others are faced with navigating a new cultural context, often finding themselves misunderstood or discriminated against, and subject to many barriers to accessing quality health care[3 4]. These challenges can endure through future generations, with many people treated like perpetual foreigners in the only home they have known.

Non-dominant ethnic groups are vulnerable to poor access to health care in several ways. A lack of familiarity with local health systems or a fear that using community resources might compromise social acceptance or immigration status can prevent people from seeking care[9]. When people from non-dominant ethnic groups do seek care, the health care provider is unlikely to share their native language or cultural heritage[10]. This can be associated with unarticulated differences in cultural beliefs about health[11] and a general breakdown in rapport or trust.[12] In the worst case, people are overtly disrespected in medical environments, compromising future health seeking behaviour.[10] Breakdowns in understanding are often magnified at a structural level. People from non-dominant ethnic groups often

For peer review only - http://bmjopen.bmj.com/site/about/guidelines.xhtml

have limited power to impact the systems around them; they are less likely to be included in decision-making structures, or to be identified as a priority group for health funding.[3]

Similar issues impact eye care.[13 14] Studies from the USA report underutilization of eye care services by non-dominant ethnic groups in general[15] and specifically by Latin Americans[16] and recent immigrants.[17] Although some public services are available (e.g. Medicaid includes eye care and some school vision screening programs) the ability to fully use services is often compromised.[13 18 19]

Some interventions exist to promote access to quality eye care for vulnerable ethnic groups[17 20 21] however these studies are diverse in terms of the population targeted, the methodological framework, and the eye problem addressed. Indeed, defining the target group is a challenge, given the difficulty in defining ethnicity and the overlap of ethnicity with socioeconomic status, education, acculturation and geography. Given this diversity, a systematic review may be inconclusive at this time, and further primary studies would not adequately build on lessons learned within this literature. A scoping review[22] appeared the most appropriate method to map and summarise this field of research.[23] This protocol (and a parallel protocol[7]) were designed to inform a project to improve access to eye care services for Māori and Pacific people in Aotearoa New Zealand, but the scope and implications are international.

Definitions and concepts

The group of primary interest in this review is difficult to define. Self-identification of ethnicity is often fluid and nuanced,[24] and appropriate terminology within health research is actively debated.[25] Although ‘ethnic minority’ is commonly used in health research to refer to a group with a shared ethnic or cultural heritage which differs from the dominant population where one resides, there is no accepted international definition.[6] For example, ’minority’ can mean numerically smaller, or it can reference lack of power or dominance.[6] Some definitions include a will to preserve a cultural identity, while others note that group membership is involuntary or imposed (this distinction is sometimes captured in the differential use of ‘ethnicity’ as self-identity vs ‘race’ as an imposed identity[25]). Indeed, many terms related to the role of ethnicity in society carry different implicit meaning across countries and time. For example, terms like ‘migrant’, ‘immigrant’ and ‘expatriate’ each reflect a new identity when living in a new place, yet differential use can reveal assumptions about perceived wealth and influence. Similarly, the terms ‘race’, ‘ethnicity’, ‘national’ and ‘visible minority’ can carry nuanced assumptions that may not be shared internationally.

Within these challenges of terminology we are interested in ethnic identities which are disempowering in their immediate context, and we refer to these groups as ‘non-dominant ethnic groups’. This could include refugees and recent immigrants as well as those who have lived in the country of residence for many generations. Since we have chosen to address Indigenous populations in a separate review,[7] our definition here is limited to people who are not Indigenous to the country in which the study is located.

For peer review only - http://bmjopen.bmj.com/site/about/guidelines.xhtml

We have defined *eye care service delivery intervention* as any organised programme or activity designed to improve access to care, according to the patient-centred access to health care framework provided by Levesque et al.[26] Levesque's framework includes a progression from health care needs to perception of needs and desire for care, to health care seeking, reaching, utilisation, and finally consequences. The progression between stages depends on services factors, including acceptability, accessibility, availability and accommodation, affordability, and appropriateness, as well as the resources and knowledge of the patient, including their ability to perceive, seek, reach, pay for and engage with health care services.

The *eye care* that will be covered will include general services (prevention and treatment services, as well as vision rehabilitation), as well as those for a particular condition or age group.

METHODS AND ANALYSIS

We have reported this protocol in accordance with the relevant sections of the PRISMA-ScR guideline[27]. The same guideline will be used to report the final review.

Scoping review questions

We aim to answer two key questions:

1) What is the extent of the published literature on interventions to promote access to eye care for non-Indigenous non-dominant ethnic groups living in high-income countries?

2) What can we learn from reported effectiveness of interventions?

3) What can we learn from authors' reflections on the potential to improve upon the interventions?

Eligibility criteria

We are interested in studies that describe interventions to improve access to eye care services for non-dominant ethnic groups (as defined above) residing in high-income countries (as defined by the World Bank[28]). This includes recent migrants, refugees and those who have resided in the high-income country for multiple generations. Given the interplay between ethnicity and socio-economic status,[29] interventions which could improve eye care services for ethnic minorities may have targeted another population (for example 'urban poor'), but had a high proportion of participants from non-dominant ethnic groups. When these studies inform our objectives, we will include studies in which at least 50% of participants are from any non-dominant, non-Indigenous population group. Given the exploratory nature of a scoping review, we will iteratively discuss this component of the inclusion criteria with an aim to include the most relevant papers and will note any changes in the final review.

For peer review only - http://bmjopen.bmj.com/site/about/guidelines.xhtml

Studies which aim to improve provision of health care more generally may include eye care e.g. interventions for diabetes care may include an assessment of diabetic retinopathy. In these cases we will only include studies if there is sufficient detail on the eye care component of the intervention to be relevant as a standalone resource. We have not limited the search to primary research projects, but chosen to include reviews, commentaries and editorials. We will include all languages and study designs, but will exclude studies for which the full text is unavailable.

Search strategy

The authors collaborated to propose relevant search terms. Final terms and strategy (details in Supplementary File 1) were then refined for use within MEDLINE, Embase and Global Health databases by Cochrane Eyes and Vision's Information Specialist (IG). An additional round of searching will be based on reference lists from included articles.

Study selection

All the results from the search will be entered into Covidence (www.covidence.org) for screening. Two authors (LMH, JR, JB, CG, RPJ or HB) will independently review each title and abstract and exclude those that do not meet the inclusion criteria. If the reviewers do not agree, the two reviewers will discuss and resolve. A third author will be consulted if no resolution can be found by the initial two reviewers. The full text of the selected articles will be reviewed, and the same two authors will independently vote to include or exclude articles. Again, conflict resolution will be handled by discussion, and a third reviewer if needed. A PRISMA flow diagram will be used to summarise the screening process.

Data charting

A data extraction form will be developed based on the data items detailed below. The form will be piloted on five studies by each of LMH, JR, JB, CG and HB, and amendments made. Given the diversity of expected results, the charting process will remain iterative, with all changes to the data charting process noted. As with the screening process, two authors will independently chart each included article. Differences in charting will be resolved through discussion, and a third author called on to resolve discrepancies if needed. If additional information is required from included studies, we will contact authors directly via email with a maximum of three attempts.

Data items

The following data items will be collected during the data charting process:

For peer review only - http://bmjopen.bmj.com/site/about/guidelines.xhtml

1. Publication characteristics: (title, year of publication, study design, country of origin, study setting)
2. Characteristics of the targeted group(s): (age, ethnicity, language, socioeconomic status, duration of residence in place of study)
3. Characteristics of the intervention (eye care context, targeted population's involvement in the development and implementation of the intervention, what was done to improve access to eye health, which dimensions of the Levesque framework for access were addressed and how).
4. Evaluated outcomes of the study (if the intervention was evaluated, how was it evaluated and what was the effectiveness, how many people were impacted by the intervention, how were baseline values and outcomes measured, and what analyses were used to draw conclusions)
5. Authors' reflections on the intervention (Authors' reflections on what worked and why, what did not and why, and any suggestions for future interventions);

Data synthesis

The interventions will be summarised descriptively and grouped according to the context, target population, eye condition(s) and access dimensions outlined above. Where interventions have been evaluated, the effectiveness, as well as identified strengths, weaknesses and suggested future directions will be summarised.

DISCUSSION

The challenges faced by some migrants in a new country can persist through many generations.[6] Access to public services, including eye care, is one such challenge.[14] The barriers are varied, influenced both by health system structures, leaders and workforce, and the resources and knowledge of the patient.[26] Given the diverse communities, with diverse barriers to eye care, varied interventions to improve access to eye care are likely to be needed. Here we have outlined a protocol for a scoping review to summarise interventions to improve access to eye care for people from non-dominant ethnic groups.

We aim to map the available literature on the topic, which may take many forms. A scoping review lends itself well to this endeavour, especially given the anticipated diversity of the work in the field[22]. The scoping review outlined here is part of a larger study to improve access to eye care services for Indigenous and non-Indigenous ethnic groups in Aotearoa New Zealand. The findings will be useful to policymakers, health service managers and clinicians responsible for eye care services in New Zealand, as well as in other countries with similar marginalised population groups. Findings will be published in an open-access peer-reviewed journal. We will also develop an accessible summary of the results for posting on institutional websites and dissemination at stakeholder meetings.

Acknowledgements: JR is a Commonwealth Rutherford Fellow, funded by the UK government through the Commonwealth Scholarship Commission in the UK. LMH is the Robert Leitl Research

For peer review only - http://bmjopen.bmj.com/site/about/guidelines.xhtml

Fellow, funded by the Robert Leitl Trust and the University of Auckland School of Optometry and Vision Science. CG holds a National Heart Foundation Fellowship.

REFERENCES

1. Braveman P. Health disparities and health equity: Concepts and measurement. Annual Review of Public Health, 2006:167-94.
2. Dawson AJ, Nkowane AM, Whelan A. Approaches to improving the contribution of the nursing and midwifery workforce to increasing universal access to primary health care for vulnerable populations: a systematic review. *Hum Resour Health* 2015;13:97. doi: 10.1186/s12960-015-0096-1
3. Abubakar I, Aldridge RW, Devakumar D, et al. The UCL-Lancet Commission on Migration and Health: the health of a world on the move. *Lancet* 2018;392(10164):2606-54. doi: 10.1016/S0140-6736(18)32114-7
4. Cooper LA, Hill MN, Powe NR. Designing and evaluating interventions to eliminate racial and ethnic disparities in health care. *J Gen Intern Med* 2002;17(6):477-86.
5. Anderson I, Robson B, Connolly M, et al. Indigenous and tribal peoples' health (The Lancet–Lowitja Institute Global Collaboration): a population study. *The Lancet* 2016;388(10040):131-57. doi: 10.1016/S0140-6736(16)00345-7
6. Indigenous peoples and ethnic minorities: Marginalization is the norm. Promoting inclusion through social protection United Nations 2018.
7. Burn H, Black J, Harwood M, et al. Eye care delivery models to improve access to eye care for Indigenous peoples in high income countries: protocol for a scoping review *BMJ Open* 2019, in press
8. Chokron S, Cavezian C, Gaudry I. Cortical visual impairment in children and their relationship with learning disorders. *ANAE - Approche Neuropsychologique des Apprentissages chez l'Enfant* 2014;26(129):117-26.
9. Hall AG. Medicaid's impact on access to and utilization of health care services among racial and ethnic minority children. *J Urban Health* 1998;75(4):677-92. doi: 10.1007/BF02344498
10. Rutledge EO. The struggle for equality in healthcare continues. *J Healthc Manag* 2001;46(5):313-24; discussion 24-6.
11. Wilson D, Heaslip V, Jackson D. Improving equity and cultural responsiveness with marginalised communities: Understanding competing worldviews. *J Clin Nurs* 2018;27(19-20):3810-19. doi: 10.1111/jocn.14546
12. Boulware LE, Cooper LA, Ratner LE, et al. Race and trust in the health care system. *Public health reports (Washington, DC : 1974)* 2003;118(4):358-65. doi: 10.1093/phr/118.4.358 [published Online First: 2003/06/20]
13. Islam NS, Kwon SC, Wyatt LC, et al. Disparities in diabetes management in Asian Americans in New York City compared with other racial/ethnic minority groups. *American Journal of Public Health* 2015;105:S443-S46. doi: 10.2105/AJPH.2014.302523
14. Varadaraj V, Frick KD, Saaddine JB, et al. Trends in Eye Care Use and Eyeglasses Affordability: The US National Health Interview Survey, 2008-2016Trends in Eye Care Use and Eyeglasses AffordabilityTrends in Eye Care Use and Eyeglasses Affordability. *JAMA Ophthalmology* 2019;137(4):391-98. doi: 10.1001/jamaophthalmol.2018.6799
15. Baker RS, Bazargan M, Bazargan-Hejazi S, et al. Access to vision care in an urban low-income multiethnic population. *Ophthalmic Epidemiol* 2005;12(1):1-12. doi: 10.1080/09286580590921330
16. Varma R, Mohanty SA, Deneen J, et al. Burden and predictors of undetected eye disease in Mexican-Americans: the Los Angeles Latino Eye Study. *Med Care* 2008;46(5):497-506. doi: 10.1097/MLR.0b013e31816080fe
17. Martin SA, Frutiger EA. Vision Stations: Addressing Corrective Vision Needs With Low-cost Technologies. *Glob Adv Health Med* 2015;4(2):46-51. doi: 10.7453/gahmj.2015.002
18. Basch CE. Vision and the achievement gap among urban minority youth. *J Sch Health* 2011;81(10):599-605. doi: 10.1111/j.1746-1561.2011.00633.x
19. Lee B. Reducing Disparities in Pediatric Vision Care Utilization by Strategically Addressing Access. *JAMA Ophthalmol* 2018 doi: 10.1001/jamaophthalmol.2018.5939
20. Walker EA, Schechter CB, Caban A, et al. Telephone intervention to promote diabetic retinopathy screening among the urban poor. *Am J Prev Med* 2008;34(3):185-91. doi: 10.1016/j.amepre.2007.11.020
21. Jones HL, Walker EA, Schechter CB, et al. Vision is precious: a successful behavioral intervention to increase the rate of screening for diabetic retinopathy for inner-city adults. *Diabetes Educ* 2010;36(1):118-26. doi: 10.1177/0145721709356116
22. Arksey H, O'Malley L. Scoping studies: towards a methodological framework. *International Journal of Social Research Methodology* 2005;8(1):19-32. doi: 10.1080/1364557032000119616
23. Munn Z, Peters MDJ, Stern C, et al. Systematic review or scoping review? Guidance for authors when choosing between a systematic or scoping review approach. *BMC Medical Research Methodology* 2018;18(1):143. doi: 10.1186/s12874-018-0611-x
24. Merino ME, Tileagă C. The construction of ethnic minority identity: A discursive psychological approach to ethnic self-definition in action. *Discourse and Society* 2011;22(1):86-101. doi: 10.1177/0957926510382834

For peer review only - http://bmjopen.bmj.com/site/about/guidelines.xhtml

25. Ford CL, Harawa NT. A new conceptualization of ethnicity for social epidemiologic and health equity research. *Social Science and Medicine* 2010;71(2):251-58. doi: 10.1016/j.socscimed.2010.04.008
26. Levesque JF, Harris MF, Russell G. Patient-centred access to health care: conceptualising access at the interface of health systems and populations. *Int J Equity Health* 2013;12:18. doi: 10.1186/1475-9276-12-18
27. Tricco AC, Lillie E, Zarin W, et al. PRISMA Extension for Scoping Reviews (PRISMA-ScR): Checklist and Explanation. *Ann Intern Med* 2018;169(7):467-73. doi: 10.7326/M18-0850
28. Watson T, Orel-Bixler D, Haegerstrom-Portnoy G. Early visual-evoked potential acuity and future behavioral acuity in cortical visual impairment. *Optometry and Vision Science* 2010;87(2):80-86. doi: 10.1097/OPX.0b013e3181c75184
29. Kawachi I, Daniels N, Robinson DER. Health Disparities By Race And Class: Why Both Matter. *Health Affairs* 2005;24(2):343-52. doi: 10.1377/hlthaff.24.2.343

For peer review only

Appendix: Search strategy

MEDLINE (Ovid)

1. exp Ophthalmology/
2. Optometry/
3. ((eye$ or ocular or vision) adj2 (care or health or service$)).tw.
4. visual acuity.tw.
5. (ophthalm$ or optomet$).tw.
6. exp Eye Diseases/
7. (glaucoma$ or ocular hypertension or cataract$).tw.
8. ((diabet$ or proliferat$) adj3 retinopath$).tw.
9. (diabet$ adj3 (eye$ or vision or visual$ or sight$)).tw.
10. (retinopath$ adj3 (eye$ or vision or visual$ or sight$)).tw.
11. (dilated adj2 fundus).tw.
12. (retinal adj2 exam$).tw.
13. (myop$ or hyperop$ or hypermetrop$ or anisometrop$ or ammetrop$ or astigmati$ or presbyop$).tw.
14. (refractive adj1 error$).tw.
15. Eyeglasses/
16. (spectacle or spectacles or glasses).tw.
17. (eyeglasses or eye glasses).tw.
18. or/1-17
19. Ethnic Groups/
20. Minority Groups/
21. ((ethnic$ or racial$ or cultur$) adj3 (group$ or minorit$ or population$ or diverse$ or origin$)).tw.
22. ((ethnic$ or racial$ or cultur$) adj3 (inequalit$ or inequit$ or disparit$ or equit$ or disadvantage$ or depriv$)).tw.
23. ((population$ or communit$) adj3 (divers$ or disadvantage$ or depriv$)).tw.
24. non-Indigenous.tw.
25. (visible adj1 minorit$).tw.
26. Refugees/
27. "emigrants and immigrants"/
28. (migrant$ or immigrant$ or emigrant$ or refugee$ or expatriate$).tw.
29. asylum seeker$.tw.
30. Urban Population/
31. urban poor.tw.
32. Cultural Characteristics/
33. Cross-Cultural Comparison/
34. Cultural Diversity/
35. Cultural competency/
36. Cultural deprivation/
37. exp African Continental Ancestry Group/
38. exp Asian Continental Ancestry Group/
39. Continental Population Groups/
40. geography.tw.
41. (Afr$ adj2 American$).tw.
42. (Afr$ adj2 Caribbean$).tw.
43. (west adj2 (india$ or indies)).tw.
44. (American$ adj1 black).tw.
45. (Asian$ or Indian$ or Pakistan$ or Bangladesh$ or Bengal$).tw.
46. ((Asia$ or Pacific) adj4 American$).tw.
47. ((Asia$ or Pacific) adj4 Islander$).tw.
48. (Hispanic or Latino or Latin American$ or Puerto Ric$ or Mexican$).tw.
49. or/19-48
50. Program Evaluation/
51. program$.tw.
52. ((educat$ or behaviour$ or behavior$ or improv$) adj3 (intervention$ or activat$)).tw.
53. Delivery of Health Care/
54. Health Services Accessibility/
55. Patient Acceptance of Health Care/
56. Health Promotion/
57. (health adj2 (promotion$ or knowledge or belief$)).tw.
58. Health Education/
59. (educat$ adj2 (information or material or leaflet)).tw.
60. Health Knowledge, Attitudes, Practice/
61. Patient Education as Topic/
62. Persuasive Communication/
63. "Surveys and Questionnaires"/
64. Questionnaires/
65. Focus Groups/
66. Health Surveys/
67. Health Care Surveys/

For peer review only - http://bmjopen.bmj.com/site/about/guidelines.xhtml

For peer review only

68. Interviews as Topic/
69. (questionnaire$ or survey$).tw.
70. (focus adj3 group$).tw.
71. exp Reminder Systems/
72. remind$.tw.
73. Telephone/
74. telephone.tw.
75. phone call.tw.
76. Health Behavior/
77. Behavior Therapy/
78. (behavioral or behavior or behaviour or behavioural).tw.
79. (increas$ adj3 (attend$ or uptake)).tw.
80. (approachability or acceptability or availability or affordability or appropriateness).tw.
81. (ability adj2 (perceive or seek or reach or pay or engage)).tw.
82. exp Vision Tests/
83. Mass Screening/
84. (vision adj3 (test$ or screen$ or assess$)).tw.
85. (eye$ adj3 (test$ or screen$ or assess$)).tw.
86. ((target$ or tailor$) adj3 intervention$).tw.
87. (cultural$ adj3 (sensitiv$ or appropriate)).tw.
88. or/50-87
89. 18 and 49 and 88
90. exp developing countries/
91. 89 not 90
92. prevalence.ti.
93. (genetic or mutation$ or autosomal or variant$).ti.
94. optical coherence tomography.tw.
95. (Latin American and Caribbean Health Sciences).tw.
96. or/92-95
97. 91 not 96
98. exp case report/
99. (case$ adj3 (report$ or series)).tw.
100. 98 or 99
101. 97 not 100

Embase (Ovid)

1. ophthalmology/
2. optometry/
3. optometrist/
4. ((eye$ or ocular or vision) adj2 (care or health or service$)).tw.
5. visual acuity.tw.
6. (ophthalm$ or optomet$).tw.
7. exp eye disease/
8. (glaucoma$ or ocular hypertension or cataract$).tw.
9. ((diabet$ or proliferat$) adj3 retinopath$).tw.
10. (diabet$ adj3 (eye$ or vision or visual$ or sight$)).tw.
11. (retinopath$ adj3 (eye$ or vision or visual$ or sight$)).tw.
12. eye fundus/
13. (dilated adj2 fundus).tw.
14. (retinal adj2 exam$).tw.
15. (myop$ or hyperop$ or hypermetrop$ or anisometrop$ or ammetrop$ or astigmati$ or presbyop$).tw.
16. (refractive adj1 error$).tw.
17. spectacles/
18. (spectacle or spectacles or glasses).tw.
19. (eyeglasses or eye glasses).tw.
20. or/1-19
21. "ethnic or racial aspects"/
22. ethnic group/
23. minority group/
24. ((ethnic$ or racial$ or cultur$) adj3 (group$ or minorit$ or population$ or diverse$ or origin$)).tw.
25. ((ethnic$ or racial$ or cultur$) adj3 (inequalit$ or inequit$ or disparit$ or equit$ or disadvantage$ or depriv$)).tw.
26. ((population$ or communit$) adj3 (divers$ or disadvantage$ or depriv$)).tw.
27. non-Indigenous.tw.
28. (visible adj1 minorit$).tw.
29. refugee/
30. asylum seeker/
31. "emigrants and immigrants"/
32. (migrant$ or immigrant$ or emigrant$ or refugee$ or expatriate$).tw.
33. asylum seeker$.tw.
34. urban population/

For peer review only - http://bmjopen.bmj.com/site/about/guidelines.xhtml

35. urban poor.tw.
36. cultural factor/
37. cultural diversity/
38. cultural competence/
39. cultural deprivation/
40. ancestry group/
41. exp african american/
42. african caribbean/
43. black person/
44. negro/
45. "Caribbean (person)"/
46. asian continental ancestry group/
47. asian american/
48. population group/
49. hispanic/
50. geography.tw.
51. (Afr$ adj2 American$).tw.
52. (Afr$ adj2 Caribbean$).tw.
53. (west adj2 (india$ or indies)).tw.
54. (American$ adj1 black).tw.
55. (Asian$ or Indian$ or Pakistan$ or Bangladesh$ or Bengal$).tw.
56. ((Asia$ or Pacific) adj4 American$).tw.
57. ((Asia$ or Pacific) adj4 Islander$).tw.
58. (Hispanic or Latino or Latin American$ or Puerto Ric$ or Mexican$).tw.
59. or/21-58
60. program evaluation/
61. program$.tw.
62. ((educat$ or behaviour$ or behavior$ or improv$) adj3 (intervention$ or activat$)).tw.
63. health care delivery/
64. patient attitude/
65. health promotion/
66. (health adj2 (promotion$ or knowledge or belief$)).tw.
67. health education/
68. (educat$ adj2 (information or material or leaflet)).tw.
69. attitude to health/
70. health behavior/
71. patient education/
72. persuasive communication/
73. questionnaires/
74. information processing/
75. health survey/
76. health care survey/
77. exp interview/
78. (questionnaire$ or survey$).tw.
79. (focus adj3 group$).tw.
80. reminder system/
81. remind$.tw.
82. telephone/
83. telephone interview/
84. telephone.tw.
85. phone call.tw.
86. health behavior/
87. behavior therapy/
88. behavior change/
89. (behavioral or behavior or behaviour or behavioural).tw.
90. (increas$ adj3 (attend$ or uptake)).tw.
91. (approachability or acceptability or availability or affordability or appropriateness).tw.
92. (ability adj2 (perceive or seek or reach or pay or engage)).tw.
93. vision test/
94. mass screening/
95. (vision adj3 (test$ or screen$ or assess$)).tw.
96. (eye$ adj3 (test$ or screen$ or assess$)).tw.
97. ((target$ or tailor$) adj3 intervention$).tw.
98. (cultural$ adj3 (sensitiv$ or appropriate)).tw.
99. or/60-98
100. 20 and 59 and 99
101. exp developing country/
102. 100 not 101
103. prevalence.ti.
104. (genetic or mutation$ or autosomal or variant$).ti.
105. optical coherence tomography.tw.
106. (Latin American and Caribbean Health Sciences).tw.
107. or/103-106
108. 102 not 107
109. exp case report/
110. (case$ adj3 (report$ or series)).tw.
111. or/109-110
112. 108 not 111
113. limit 112 to conference abstract status
114. 112 not 113

For peer review only

Global Health (Ovid)

1. eyes/
2. eye diseases/
3. vision/
4. vision disorders/
5. ((eye$ or ocular or vision) adj2 (care or health or service$)).tw.
6. visual acuity.tw.
7. (ophthalm$ or optomet$).tw.
8. (glaucoma$ or ocular hypertension or cataract$).tw.
9. ((diabet$ or proliferat$) adj3 retinopath$).tw.
10. (diabet$ adj3 (eye$ or vision or visual$ or sight$)).tw.
11. (retinopath$ adj3 (eye$ or vision or visual$ or sight$)).tw.
12. (dilated adj2 fundus).tw.
13. (retinal adj2 exam$).tw.
14. (myop$ or hyperop$ or hypermetrop$ or anisometrop$ or ammetrop$ or astigmati$ or presbyop$).tw.
15. (refractive adj1 error$).tw.
16. (spectacle or spectacles or glasses).tw.
17. (eyeglasses or eye glasses).tw.
18. or/1-17
19. ethnic groups/
20. ethnicity/
21. minorities/
22. ((ethnic$ or racial$ or cultur$) adj3 (group$ or minorit$ or population$ or diverse$ or origin$)).tw.
23. ((ethnic$ or racial$ or cultur$) adj3 (inequalit$ or inequit$ or disparit$ or equit$ or disadvantage$ or depriv$)).tw.
24. ((population$ or communit$) adj3 (divers$ or disadvantage$ or depriv$)).tw.
25. non-Indigenous.tw.
26. (visible adj1 minorit$).tw.
27. refugees/
28. immigrants/
29. immigration/
30. (migrant$ or immigrant$ or emigrant$ or refugee$ or expatriate$).tw.
31. asylum seeker$.tw.
32. urban population/
33. urban areas/
34. urban poor.tw.
35. geography.tw.
36. african americans/ or african-caribbeans/ or american indians/ or asians/ or black people/ or japanese americans/ or mexican-americans/ or pacific islanders/
37. (Afr$ adj2 American$).tw.
38. (Afr$ adj2 Caribbean$).tw.
39. (west adj2 (india$ or indies)).tw.
40. (American$ adj1 black).tw.
41. (Asian$ or Indian$ or Pakistan$ or Bangladesh$ or Bengal$).tw.
42. ((Asia$ or Pacific) adj4 American$).tw.
43. or/19-42
44. program evaluation/
45. programs/
46. program$.tw.
47. ((educat$ or behaviour$ or behavior$ or improv$) adj3 (intervention$ or activat$)).tw.
48. (primary health care or health services or community health or health policy or health care or public health or health programmes).sh.
49. health promotion/
50. (health adj2 (promotion$ or knowledge or belief$)).tw.
51. health education/
52. patient education/
53. (educat$ adj2 (information or material or leaflet)).tw.
54. attitude/
55. communication/
56. questionnaires/
57. discussion groups/
58. surveys/
59. (questionnaire$ or survey$).tw.
60. (focus adj3 group$).tw.
61. remind$.tw.
62. telephone/
63. telephone.tw.
64. phone call.tw.

For peer review only - http://bmjopen.bmj.com/site/about/guidelines.xhtml

65. behaviour/
66. (behavioral or behavior or behaviour or behavioural).tw.
67. (increas$ adj3 (attend$ or uptake)).tw.
68. (approachability or acceptability or availability or affordability or appropriateness).tw.
69. (ability adj2 (perceive or seek or reach or pay or engage)).tw.
70. screening/
71. (vision adj3 (test$ or screen$ or assess$)).tw.
72. (eye$ adj3 (test$ or screen$ or assess$)).tw.
73. ((target$ or tailor$) adj3 intervention$).tw.
74. (cultural$ adj3 (sensitiv$ or appropriate)).tw.
75. culture/
76. or/44-75
77. 18 and 43 and 76
78. exp developing countries/
79. 77 not 78
80. prevalence.ti.
81. (genetic or mutation$ or autosomal or variant$).ti.
82. optical coherence tomography.tw.
83. (Latin American and Caribbean Health Sciences).tw.
84. or/80-83
85. 79 not 84
86. exp case report/
87. (case$ adj3 (report$ or series)).tw.
88. 86 or 87
89. 85 not 88

For peer review only - http://bmjopen.bmj.com/site/about/guidelines.xhtml

BMJ Open

Interventions to promote access to eye care for non-Indigenous, non-dominant ethnic groups in high-income countries: a scoping review protocol

Journal:	*BMJ Open*
Manuscript ID	bmjopen-2019-033775.R1
Article Type:	Protocol
Date Submitted by the Author:	02-Apr-2020

Complete List of Authors:	Hamm, Lisa; The University of Auckland, School of Optometry and Vision Science<br>Black, Joanna; The University of Auckland, School of Optometry and Vision Science<br>Burn, Helen; Brighton and Sussex University Hospitals NHS Trust<br>Grey, Corina; University of Auckland, School of Population Health<br>Harwood, Matire; University of Auckland, School of Population Health<br>Peiris-John, Roshini; The University of Auckland, School of Population Health<br>Gordon, Iris; London School of Hygiene and Tropical Medicine Faculty of Infectious and Tropical Diseases, International Centre for Eye Health<br>Burton, Matthew J; London School of Hygiene and Tropical Medicine<br>Evans, Jennifer; London School of Hygiene and Tropical Medicine Faculty of Infectious and Tropical Diseases, International Centre for Eye Health<br>Ramke, Jacqueline; London School of Hygiene and Tropical Medicine Faculty of Infectious and Tropical Diseases, International Centre for Eye Health; The University of Auckland, School of Optometry and Vision Science

**Primary Subject Heading**:	Ophthalmology
Secondary Subject Heading:	Global health, Health services research, Patient-centred medicine
Keywords:	ethnic minority, ethnic disparity, immigrants, eye care, optometry

SCHOLARONE™ Manuscripts

I, the Submitting Author has the right to grant and does grant on behalf of all authors of the Work (as defined in the below author licence), an exclusive licence and/or a non-exclusive licence for contributions from authors who are: i) UK Crown employees; ii) where BMJ has agreed a CC-BY licence shall apply, and/or iii) in accordance with the terms applicable for US Federal Government officers or employees acting as part of their official duties; on a worldwide, perpetual, irrevocable, royalty-free basis to BMJ Publishing Group Ltd ("BMJ") its licensees and where the relevant Journal is co-owned by BMJ to the co-owners of the Journal, to publish the Work in this journal and any other BMJ products and to exploit all rights, as set out in our licence.

The Submitting Author accepts and understands that any supply made under these terms is made by BMJ to the Submitting Author unless you are acting as an employee on behalf of your employer or a postgraduate student of an affiliated institution which is paying any applicable article publishing charge ("APC") for Open Access articles. Where the Submitting Author wishes to make the Work available on an Open Access basis (and intends to pay the relevant APC), the terms of reuse of such Open Access shall be governed by a Creative Commons licence – details of these licences and which Creative Commons licence will apply to this Work are set out in our licence referred to above.

Other than as permitted in any relevant BMJ Author's Self Archiving Policies, I confirm this Work has not been accepted for publication elsewhere, is not being considered for publication elsewhere and does not duplicate material already published. I confirm all authors consent to publication of this Work and authorise the granting of this licence.

For peer review only

Interventions to promote access to eye care for non-Indigenous, non-dominant ethnic groups in high-income countries: a scoping review protocol

Lisa M **Hamm**[1]	l.hamm@auckland.ac.nz	0000-0003-2777-7146
Joanna **Black**[1]	j.black@auckland.ac.nz	0000-0002-5100-8796
Helen **Burn**[2]	hburn88@gmail.com	0000-0002-1469-8169
Corina **Grey**[3]	c.grey@auckland.ac.nz	0000-0003-1751-1934
Matire **Harwood**[3]	m.harwood@auckland.ac.nz	0000-0003-1240-5139
Roshini **Peiris-John**[3]	r.peiris-john@auckland.ac.nz	0000-0001-7812-2268
Iris **Gordon**[2]	Iris.Gordon@lshtm.ac.uk	0000-0001-8143-8132
Matthew J **Burton**[2,4]	Matthew.Burton@lshtm.ac.uk	0000-0003-1872-9169
Jennifer R **Evans**[2]	Jennifer.Evans@lshtm.ac.uk	0000-0002-6137-2030
Jacqueline **Ramke**[1,2]	Jacqueline.Ramke@lshtm.ac.uk	0000-0002-5764-1306

1. School of Optometry & Vision Science, Faculty of Medicine and Health Sciences, The University of Auckland, Auckland, New Zealand
2. International Centre for Eye Health, London School of Hygiene and Tropical Medicine, London, United Kingdom
3. School of Population Health, Faculty of Medicine and Health Sciences, University of Auckland, Auckland, New Zealand
4. Moorfields Eye Hospital, London, United Kingdom

Author Contributors: JR conceived the idea for the review. LMH and JR drafted and revised the protocol with suggestions from JB, HB, CG, MH, RPJ, MJB and JE who reviewed the protocol and provided feedback on the draft. IG constructed the search.

Corresponding Author: Jacqueline Ramke (jacqueline.ramke@lshtm.ac.uk)

Funding Statement: This work was supported by The University of Auckland Faculty Research Development Fund grant number 3716758. JR is a Commonwealth Rutherford Fellow, funded by the UK government through the Commonwealth Scholarship Commission in the UK. LMH is the Robert Leitl Research Fellow, funded by the Robert Leitl Trust and the University of Auckland School of Optometry and Vision Science. CG holds a National Heart Foundation Fellowship. MJB is supported by the Wellcome Trust (207472/Z/17/Z).

Competing Interests: None declared. **Table Number**: 0. **Figure Number**: 1

Patient and Public Involvement Statement: As this was a review of existing literature, this research was done without specific patient involvement.

Data Sharing Statement: Data generated from this review will be available upon reasonable request from Jacqueline Ramke (jacqueline.ramke@lshtm.ac.uk)

Keywords: non-dominant ethnic group, ethnic minority, ethnic disparity, immigrants, eye care, optometry, ophthalmology, access, service delivery, health

Word count: 2240

For peer review only - http://bmjopen.bmj.com/site/about/guidelines.xhtml

Strengths and limitations of this study

- This study will provide a comprehensive overview of the published literature on interventions to improve access to eye care for non-Indigenous, non-dominant ethnic groups in high-income countries.
- The review will be comprehensive, including published literature of all study designs, without time period or language restrictions.
- A potential limitation could be a paucity of information on the topic.
- Another potential limitation is that the population of interest is difficult to define.

For peer review only

For peer review only - http://bmjopen.bmj.com/site/about/guidelines.xhtml

ABSTRACT

Introduction

For many people, settling in a new country is associated with a new identity as an 'ethnic minority', one that can remain through future generations. People who are culturally distinct from the dominant population group may experience a variety of barriers to accessing health care, including linguistic and cultural barriers in communication, navigation of an unfamiliar health system, and unconscious or overt discrimination. Here we outline the protocol of a scoping review to identify, describe and summarise interventions aimed at improving access to eye care for non-Indigenous, non-dominant ethnic groups residing in high-income countries.

Methods and analysis

We will search MEDLINE, Embase and Global Health from their inception with no date limits. We will include studies of any design that describe an intervention to promote access to eye care for non-Indigenous, non-dominant ethnic groups. Two authors will independently review titles, abstracts, and full-text articles for inclusion. Reference lists from all included articles will also be searched. In cases of disagreement between initial reviewers, a third author will help resolve the conflict. For each included article we will extract data about the target population, details of the intervention delivered and the effectiveness of or feedback from the intervention. Overall findings will be summarised with descriptive statistics and thematic analysis.

Ethics and dissemination

This review will summarise existing literature and as such ethics approval is not required. We will publish the review in an open-access, peer-reviewed journal, and draft appropriate summaries for dissemination to the wider community. This wider community could include clinicians, policymakers, health service managers and organisations that work with vulnerable ethnic minorities. Our findings will also feed into the ongoing *Lancet Global Health* Commission on Global Eye Health.

For peer review only

INTRODUCTION

Rationale

Equitable access to health care is critically important, but it is a challenge to both define and achieve.[1] Health systems are often implicitly structured to meet the needs and preferences of members of the dominant group in any given population, which makes these systems more challenging for people with diverse backgrounds to navigate.[2 3] The axes of diversity vary widely (including socio-economic status, gender, sexual orientation, Indigeneity) and are often intersectional. Challenges in navigation of health care systems are compounded for people with a non-dominant ethnic background, because the health care seeker is more likely to look, speak, and communicate differently to their health care providers.[4]

The history of each ethnic group in a given place can influence how and to what extent health services strive to mitigate the vulnerabilities experienced by the group. For example, there is increasing recognition of overt institutional racism against colonised Indigenous populations and the impact this has on health care and health outcomes.[5] Formal efforts at restitution[6] have attempted to improve access to health care such as government-funded services to rural and remote areas with high Indigenous populations, and health facilities within Indigenous communities. For this reason, we are investigating service delivery models to improve access to eye care for Indigenous populations in a complementary scoping review,[7] and in the scoping review outlined here, we consider interventions to promote access to eye care for *non-Indigenous*, non-dominant ethnic groups.

A 'migrant' is a person who is living or has lived in a different place than they were born.[3] Using this definition, it was estimated that 3.4% of the global population (258 million people) were migrants in 2017.[8] People move away from their country of birth for a variety of reasons; many move for employment, others are forced from their home country because of civil unrest or violence, and others are moved through human trafficking and modern slavery.[3] Some migrants arrive in countries with a similar culture and language to their own, while others are faced with navigating a new cultural context, often finding themselves misunderstood or discriminated against, and subject to many barriers to accessing quality health care.[3 4] These challenges can endure through future generations, with many people treated like perpetual foreigners in the only home they have known.

Non-dominant ethnic groups are vulnerable to poor access to health care in several ways. A lack of familiarity with local health systems or a fear that using community resources might compromise social acceptance or immigration status can prevent people from seeking care.[9] When people from non-dominant ethnic groups do seek care, the health care provider is unlikely to share their native language or cultural heritage.[10] This can be associated with unarticulated differences in cultural beliefs about health[11] and a general breakdown in rapport or trust.[12] In the worst case, people are overtly disrespected in medical environments, compromising future health seeking behaviour.[10] Breakdowns in understanding are often magnified at a structural level. People from non-dominant

For peer review only - http://bmjopen.bmj.com/site/about/guidelines.xhtml

ethnic groups often have limited power to impact the systems around them; they are less likely to be included in decision-making structures, or to be identified as a priority group for health funding.[3]

Similar issues impact eye care.[13 14] Studies from the USA report underutilization of eye care services by non-dominant ethnic groups in general[15] and specifically by Latin Americans[16] and recent immigrants.[17] Although some public services are available (e.g. Medicaid includes eye care and some school vision screening programs) the ability to fully use services is often compromised.[13 18 19]

Some interventions exist to promote access to quality eye care for vulnerable ethnic groups[17 20 21] however these studies are diverse in terms of the population targeted, the methodological framework, and the eye problem addressed. Indeed, defining the target group is a challenge, given the difficulty in defining ethnicity and the overlap of ethnicity with socioeconomic status, education, acculturation and geography. Given this diversity, a systematic review may be inconclusive at this time, and further primary studies would not adequately build on lessons learned within this literature. A scoping review[22] appeared the most appropriate method to map and summarise this field of research.[23] This protocol (and a parallel protocol[7]) were designed to inform a project to improve access to eye care services for Māori and Pacific people in Aotearoa New Zealand, but the scope and implications are international.

Definitions and concepts

The group of primary interest in this review is difficult to define. Self-identification of ethnicity is often fluid and nuanced,[24] and appropriate terminology within health research is actively debated.[25] Although 'ethnic minority' is commonly used in health research to refer to a group with a shared ethnic or cultural heritage which differs from the dominant population where one resides, there is no accepted international definition.[6] For example, 'minority' can mean numerically smaller, or it can reference lack of power or dominance.[6] Some definitions include a will to preserve a cultural identity, while others note that group membership is involuntary or imposed (this distinction is sometimes captured in the differential use of 'ethnicity' as self-identity vs 'race' as an imposed identity[25]). Indeed, many terms related to the role of ethnicity in society carry different implicit meaning across countries and time. For example, terms like 'migrant', 'immigrant' and 'expatriate' each reflect a new identity when living in a new place, yet differential use can reveal assumptions about perceived wealth and influence. Similarly, the terms 'race', 'ethnicity', 'national' and 'visible minority' can carry nuanced assumptions that may not be shared internationally.

Within these challenges of terminology, we are interested in ethnic identities which are disempowering in their immediate context, and we refer to these groups as 'non-dominant ethnic groups'. This could include refugees and recent immigrants as well as those who have lived in the country of residence for many generations. Since we have chosen to address Indigenous populations in a separate review,[7] our definition here is limited to people who are not Indigenous to the country in which the study is located.

For peer review only - http://bmjopen.bmj.com/site/about/guidelines.xhtml

We have defined *eye care service delivery intervention* as any organised programme or activity designed to improve access to care, according to the patient-centred access to health care framework provided by Levesque et al.[26] Levesque's framework includes a progression from health care needs to perception of needs and desire for care, to health care seeking, reaching, utilisation, and finally consequences (Figure 1). The progression between stages depends on services factors, including acceptability, accessibility, availability and accommodation, affordability, and appropriateness, as well as the resources and knowledge of the patient, including their ability to perceive, seek, reach, pay for and engage with health care services.

The *eye care* that will be covered will include general services (prevention and treatment services, as well as vision rehabilitation), as well as those for a particular condition or age group.

Insert Figure 1 here

Figure 1: Conceptual framework for access to healthcare (reproduced from Levesque *et al.* [26]*)*

METHODS AND ANALYSIS

We have reported this protocol in accordance with the relevant sections of the PRISMA-ScR guideline.[27] The same guideline will be used to report the final review.

Scoping review questions

We aim to answer three key questions:

1) What is the extent of the published literature on interventions to promote access to eye care for non-Indigenous non-dominant ethnic groups living in high-income countries?

2) What can we learn from reported effectiveness of interventions?

3) What can we learn from authors' reflections on the potential to improve upon the interventions?

Eligibility criteria

We are interested in studies that describe interventions to improve access to eye care services for non-dominant ethnic groups (as defined above) residing in high-income countries (as defined by the World Bank[28]). This includes recent migrants, refugees and those who have resided in the high-income country for multiple generations. Given the interplay between ethnicity and socio-economic status,[29] interventions which could improve eye care services for ethnic minorities may have targeted another population (for example 'urban poor'), but had a high proportion of participants from non-dominant ethnic groups. When these studies inform our objectives, we will include studies in which at least 50% of participants are from any non-dominant, non-Indigenous population group. Given the exploratory nature of a scoping review, we will iteratively discuss this component of the inclusion criteria with an aim to include the most relevant papers and will note any changes in the final review.

For peer review only - http://bmjopen.bmj.com/site/about/guidelines.xhtml

For peer review only

Studies which aim to improve provision of health care more generally may include eye care e.g. interventions for diabetes care may include an assessment of diabetic retinopathy. In these cases, we will only include studies if there is sufficient detail on the eye care component of the intervention to be relevant as a standalone resource. We will exclude reviews, commentaries and editorials, but will check the reference list of review articles for potentially relevant studies. . We will include all languages and study designs, but will exclude studies for which the full text is unavailable after exhausting university library resources.

Search strategy

The authors collaborated to propose relevant search terms. Final terms and strategy (details in Supplementary File 1) were then refined for use within MEDLINE, Embase and Global Health databases by Cochrane Eyes and Vision's Information Specialist (IG). An additional round of searching will be based on reference lists from included articles and relevant reviews.

Study selection

All the results from the search will be entered into Covidence (www.covidence.org) for screening. Two authors (from LMH, JR, JB, CG, RPJ or HB) will independently review each title and abstract and exclude those that do not meet the inclusion criteria. If the reviewers do not agree, the two reviewers will discuss and resolve. A third author will be consulted if no resolution can be found by the initial two reviewers. The full text of the selected articles will be reviewed, and the same two authors will independently vote to include or exclude articles. Again, conflict resolution will be handled by discussion, and a third reviewer if needed. A PRISMA flow diagram will be used to summarise the screening process.

Data charting

A data extraction form will be developed based on the data items detailed below. The form will be piloted on five studies by each of LMH, JR, JB, CG and HB, and amendments made. Given the diversity of expected results, the charting process will remain iterative, with all changes to the data charting process noted. As with the screening process, two authors will independently chart each included article. Differences in charting will be resolved through discussion, and a third author called on to resolve discrepancies if needed. If additional information is required from included studies, we will contact authors directly via email with a maximum of three attempts.

Data items

The following data items will be collected during the data charting process:

1. Publication characteristics: (title, year of publication, study design, country of origin, study setting)
2. Characteristics of the targeted group(s): (age, ethnicity, language, socioeconomic status, duration of residence in place of study)

For peer review only - http://bmjopen.bmj.com/site/about/guidelines.xhtml

3. Characteristics of the intervention (eye care context, targeted population's involvement in the development and implementation of the intervention, what was done to improve access to eye health, which dimensions of the Levesque framework for access were addressed and how).
4. Evaluated outcomes of the study (if the intervention was evaluated, how was it evaluated and what was the effectiveness, how many people were impacted by the intervention, how were baseline values and outcomes measured, and what analyses were used to draw conclusions)
5. Authors' reflections on the intervention (Authors' reflections on what worked and why, what did not and why, and any suggestions for future interventions).

Data synthesis

The interventions will be summarised descriptively and grouped according to the context, target population, eye condition(s) and access dimensions outlined above. Where interventions have been evaluated, the effectiveness, as well as identified strengths, weaknesses and suggested future directions will be summarised.

Ethics and dissemination

This review will summarise existing literature and as such ethics approval is not required. We will publish the review in an open-access, peer-reviewed journal, and draft appropriate summaries for dissemination to the wider community. This wider community could include clinicians, policymakers, health service managers and organisations that work with vulnerable ethnic minorities. Our findings will also feed into the ongoing *Lancet Global Health* Commission on Global Eye Health.[30]

DISCUSSION

The challenges faced by some migrants in a new country can persist through many generations.[6] Access to public services, including eye care, is one such challenge.[14] The barriers are varied, influenced both by health system structures, leaders and workforce, and the resources and knowledge of the patient.[26] Given the diverse communities, with diverse barriers to eye care, varied interventions to improve access to eye care are likely to be needed. Here we have outlined a protocol for a scoping review to summarise interventions to improve access to eye care for people from non-dominant ethnic groups.

We aim to map the available literature on the topic, which may take many forms. A scoping review lends itself well to this endeavour, especially given the anticipated diversity of the work in the field.[22] The scoping review outlined here is part of a larger study to improve access to eye care services for Indigenous and non-Indigenous ethnic groups in Aotearoa New Zealand. The findings will be useful to policymakers, health service managers and clinicians responsible for eye care services in New Zealand, as well as in other countries with similar marginalised population groups. In addition to publication in an open access journal, we will develop an accessible summary of the results for posting on institutional websites and dissemination at stakeholder meetings.

For peer review only - http://bmjopen.bmj.com/site/about/guidelines.xhtml

REFERENCES

1. Braveman P. Health disparities and health equity: Concepts and measurement. *Annual Review of Public Health*, 2006:167-94.
2. Dawson AJ, Nkowane AM, Whelan A. Approaches to improving the contribution of the nursing and midwifery workforce to increasing universal access to primary health care for vulnerable populations: a systematic review. *Hum Resour Health* 2015;13:97. doi: 10.1186/s12960-015-0096-1
3. Abubakar I, Aldridge RW, Devakumar D, et al. The UCL-Lancet Commission on Migration and Health: the health of a world on the move. *Lancet* 2018;392(10164):2606-54. doi: 10.1016/S0140-6736(18)32114-7
4. Cooper LA, Hill MN, Powe NR. Designing and evaluating interventions to eliminate racial and ethnic disparities in health care. *J Gen Intern Med* 2002;17(6):477-86.
5. Anderson I, Robson B, Connolly M, et al. Indigenous and tribal peoples' health (The Lancet–Lowitja Institute Global Collaboration): a population study. *The Lancet* 2016;388(10040):131-57. doi: 10.1016/S0140-6736(16)00345-7
6. Indigenous peoples and ethnic minorities: Marginalization is the norm. Promoting inclusion through social protection United Nations 2018.
7. Burn H, Black J, Harwood M, et al. Eye care delivery models to improve access to eye care for Indigenous peoples in high income countries: protocol for a scoping review *BMJ Open* 2019, in press
8. Chokron S, Cavezian C, Gaudry I. Cortical visual impairment in children and their relationship with learning disorders. *ANAE - Approche Neuropsychologique des Apprentissages chez l'Enfant* 2014;26(129):117-26.
9. Hall AG. Medicaid's impact on access to and utilization of health care services among racial and ethnic minority children. *J Urban Health* 1998;75(4):677-92. doi: 10.1007/BF02344498
10. Rutledge EO. The struggle for equality in healthcare continues. *J Healthc Manag* 2001;46(5):313-24; discussion 24-6.
11. Wilson D, Heaslip V, Jackson D. Improving equity and cultural responsiveness with marginalised communities: Understanding competing worldviews. *J Clin Nurs* 2018;27(19-20):3810-19. doi: 10.1111/jocn.14546
12. Boulware LE, Cooper LA, Ratner LE, et al. Race and trust in the health care system. *Public health reports (Washington, DC : 1974)* 2003;118(4):358-65. doi: 10.1093/phr/118.4.358 [published Online First: 2003/06/20]
13. Islam NS, Kwon SC, Wyatt LC, et al. Disparities in diabetes management in Asian Americans in New York City compared with other racial/ethnic minority groups. *American Journal of Public Health* 2015;105:S443-S46. doi: 10.2105/AJPH.2014.302523
14. Varadaraj V, Frick KD, Saaddine JB, et al. Trends in Eye Care Use and Eyeglasses Affordability: The US National Health Interview Survey, 2008-2016Trends in Eye Care Use and Eyeglasses AffordabilityTrends in Eye Care Use and Eyeglasses Affordability. *JAMA Ophthalmology* 2019;137(4):391-98. doi: 10.1001/jamaophthalmol.2018.6799
15. Baker RS, Bazargan M, Bazargan-Hejazi S, et al. Access to vision care in an urban low-income multiethnic population. *Ophthalmic Epidemiol* 2005;12(1):1-12. doi: 10.1080/09286580590921330
16. Varma R, Mohanty SA, Deneen J, et al. Burden and predictors of undetected eye disease in Mexican-Americans: the Los Angeles Latino Eye Study. *Med Care* 2008;46(5):497-506. doi: 10.1097/MLR.0b013e31816080fe
17. Martin SA, Frutiger EA. Vision Stations: Addressing Corrective Vision Needs With Low-cost Technologies. *Glob Adv Health Med* 2015;4(2):46-51. doi: 10.7453/gahmj.2015.002
18. Basch CE. Vision and the achievement gap among urban minority youth. *J Sch Health* 2011;81(10):599-605. doi: 10.1111/j.1746-1561.2011.00633.x
19. Lee B. Reducing Disparities in Pediatric Vision Care Utilization by Strategically Addressing Access. *JAMA Ophthalmol* 2018 doi: 10.1001/jamaophthalmol.2018.5939
20. Walker EA, Schechter CB, Caban A, et al. Telephone intervention to promote diabetic retinopathy screening among the urban poor. *Am J Prev Med* 2008;34(3):185-91. doi: 10.1016/j.amepre.2007.11.020
21. Jones HL, Walker EA, Schechter CB, et al. Vision is precious: a successful behavioral intervention to increase the rate of screening for diabetic retinopathy for inner-city adults. *Diabetes Educ* 2010;36(1):118-26. doi: 10.1177/0145721709356116
22. Arksey H, O'Malley L. Scoping studies: towards a methodological framework. *International Journal of Social Research Methodology* 2005;8(1):19-32. doi: 10.1080/1364557032000119616

For peer review only - http://bmjopen.bmj.com/site/about/guidelines.xhtml

23. Munn Z, Peters MDJ, Stern C, et al. Systematic review or scoping review? Guidance for authors when choosing between a systematic or scoping review approach. *BMC Medical Research Methodology* 2018;18(1):143. doi: 10.1186/s12874-018-0611-x
24. Merino ME, Tileagă C. The construction of ethnic minority identity: A discursive psychological approach to ethnic self-definition in action. *Discourse and Society* 2011;22(1):86-101. doi: 10.1177/0957926510382834
25. Ford CL, Harawa NT. A new conceptualization of ethnicity for social epidemiologic and health equity research. *Social Science and Medicine* 2010;71(2):251-58. doi: 10.1016/j.socscimed.2010.04.008
26. Levesque JF, Harris MF, Russell G. Patient-centred access to health care: conceptualising access at the interface of health systems and populations. *Int J Equity Health* 2013;12:18. doi: 10.1186/1475-9276-12-18
27. Tricco AC, Lillie E, Zarin W, et al. PRISMA Extension for Scoping Reviews (PRISMA-ScR): Checklist and Explanation. *Ann Intern Med* 2018;169(7):467-73. doi: 10.7326/M18-0850
28. Watson T, Orel-Bixler D, Haegerstrom-Portnoy G. Early visual-evoked potential acuity and future behavioral acuity in cortical visual impairment. *Optometry and Vision Science* 2010;87(2):80-86. doi: 10.1097/OPX.0b013e3181c75184
29. Kawachi I, Daniels N, Robinson DER. Health Disparities By Race And Class: Why Both Matter. *Health Affairs* 2005;24(2):343-52. doi: 10.1377/hlthaff.24.2.343
30. Burton MJ, Faal HB, Ramke J, et al. Announcing The Lancet Global Health Commission on Global Eye Health. *The Lancet Global Health* 2019;7(12):e1612-e13. doi: 10.1016/S2214-109X(19)30450-4

For peer review only

For peer review only - http://bmjopen.bmj.com/site/about/guidelines.xhtml

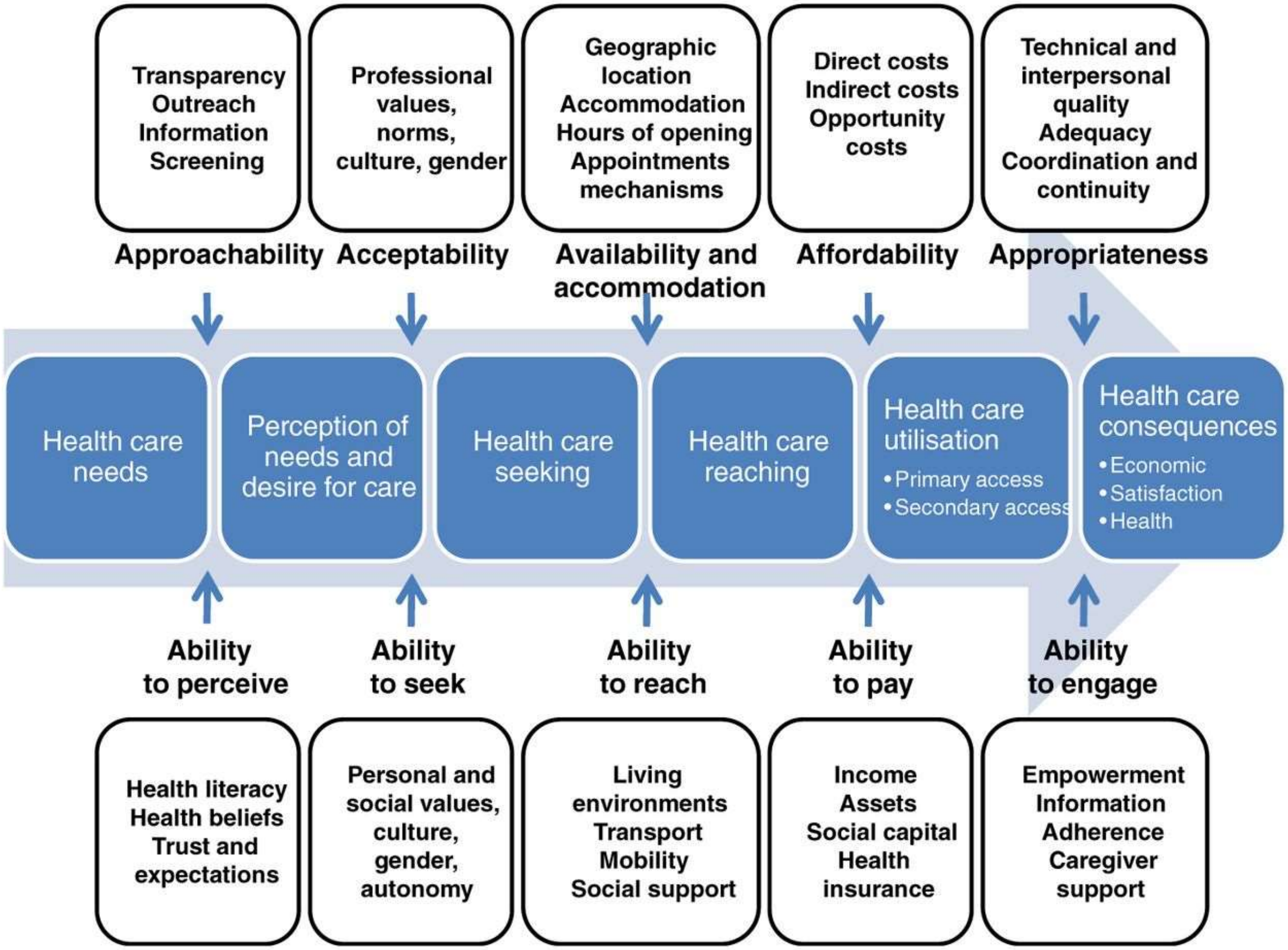
Transparency
Outreach
Information
Screening
Professional values, norms, culture, gender
Geographic location
Accommodation
Hours of opening
Appointments mechanisms
Direct costs
Indirect costs
Opportunity costs
Technical and interpersonal quality
Adequacy
Coordination and continuity
Approachability
Acceptability
Availability and accommodation
Affordability
Appropriateness
Health care needs
Perception of needs and desire for care
Health care seeking
Health care reaching
Health care utilisation
• Primary access
• Secondary access
Health care consequences
• Economic
• Satisfaction
• Health
Ability to perceive
Ability to seek
Ability to reach
Ability to pay
Ability to engage
Health literacy
Health beliefs
Trust and expectations
Personal and social values, culture, gender, autonomy
Living environments
Transport
Mobility
Social support
Income
Assets
Social capital
Health insurance
Empowerment
Information
Adherence
Caregiver support

Appendix: Search strategy

MEDLINE (Ovid)

1. exp Ophthalmology/
2. Optometry/
3. ((eye$ or ocular or vision) adj2 (care or health or service$)).tw.
4. visual acuity.tw.
5. (ophthalm$ or optomet$).tw.
6. exp Eye Diseases/
7. (glaucoma$ or ocular hypertension or cataract$).tw.
8. ((diabet$ or proliferat$) adj3 retinopath$).tw.
9. (diabet$ adj3 (eye$ or vision or visual$ or sight$)).tw.
10. (retinopath$ adj3 (eye$ or vision or visual$ or sight$)).tw.
11. (dilated adj2 fundus).tw.
12. (retinal adj2 exam$).tw.
13. (myop$ or hyperop$ or hypermetrop$ or anisometrop$ or ammetrop$ or astigmati$ or presbyop$).tw.
14. (refractive adj1 error$).tw.
15. Eyeglasses/
16. (spectacle or spectacles or glasses).tw.
17. (eyeglasses or eye glasses).tw.
18. or/1-17
19. Ethnic Groups/
20. Minority Groups/
21. ((ethnic$ or racial$ or cultur$) adj3 (group$ or minorit$ or population$ or diverse$ or origin$)).tw.
22. ((ethnic$ or racial$ or cultur$) adj3 (inequalit$ or inequit$ or disparit$ or equit$ or disadvantage$ or depriv$)).tw.
23. ((population$ or communit$) adj3 (divers$ or disadvantage$ or depriv$)).tw.
24. non-Indigenous.tw.
25. (visible adj1 minorit$).tw.
26. Refugees/
27. "emigrants and immigrants"/
28. (migrant$ or immigrant$ or emigrant$ or refugee$ or expatriate$).tw.
29. asylum seeker$.tw.
30. Urban Population/
31. urban poor.tw.
32. Cultural Characteristics/
33. Cross-Cultural Comparison/
34. Cultural Diversity/
35. Cultural competency/
36. Cultural deprivation/
37. exp African Continental Ancestry Group/
38. exp Asian Continental Ancestry Group/
39. Continental Population Groups/
40. geography.tw.
41. (Afr$ adj2 American$).tw.
42. (Afr$ adj2 Caribbean$).tw.
43. (west adj2 (india$ or indies)).tw.
44. (American$ adj1 black).tw.
45. (Asian$ or Indian$ or Pakistan$ or Bangladesh$ or Bengal$).tw.
46. ((Asia$ or Pacific) adj4 American$).tw.
47. ((Asia$ or Pacific) adj4 Islander$).tw.
48. (Hispanic or Latino or Latin American$ or Puerto Ric$ or Mexican$).tw.
49. or/19-48
50. Program Evaluation/
51. program$.tw.
52. ((educat$ or behaviour$ or behavior$ or improv$) adj3 (intervention$ or activat$)).tw.
53. Delivery of Health Care/
54. Health Services Accessibility/
55. Patient Acceptance of Health Care/
56. Health Promotion/
57. (health adj2 (promotion$ or knowledge or belief$)).tw.
58. Health Education/
59. (educat$ adj2 (information or material or leaflet)).tw.
60. Health Knowledge, Attitudes, Practice/
61. Patient Education as Topic/
62. Persuasive Communication/
63. "Surveys and Questionnaires"/
64. Questionnaires/
65. Focus Groups/
66. Health Surveys/
67. Health Care Surveys/

For peer review only - http://bmjopen.bmj.com/site/about/guidelines.xhtml

68. Interviews as Topic/
69. (questionnaire$ or survey$).tw.
70. (focus adj3 group$).tw.
71. exp Reminder Systems/
72. remind$.tw.
73. Telephone/
74. telephone.tw.
75. phone call.tw.
76. Health Behavior/
77. Behavior Therapy/
78. (behavioral or behavior or behaviour or behavioural).tw.
79. (increas$ adj3 (attend$ or uptake)).tw.
80. (approachability or acceptability or availability or affordability or appropriateness).tw.
81. (ability adj2 (perceive or seek or reach or pay or engage)).tw.
82. exp Vision Tests/
83. Mass Screening/
84. (vision adj3 (test$ or screen$ or assess$)).tw.
85. (eye$ adj3 (test$ or screen$ or assess$)).tw.
86. ((target$ or tailor$) adj3 intervention$).tw.
87. (cultural$ adj3 (sensitiv$ or appropriate)).tw.
88. or/50-87
89. 18 and 49 and 88
90. exp developing countries/
91. 89 not 90
92. prevalence.ti.
93. (genetic or mutation$ or autosomal or variant$).ti.
94. optical coherence tomography.tw.
95. (Latin American and Caribbean Health Sciences).tw.
96. or/92-95
97. 91 not 96
98. exp case report/
99. (case$ adj3 (report$ or series)).tw.
100. 98 or 99
101. 97 not 100

Embase (Ovid)

1. ophthalmology/
2. optometry/
3. optometrist/
4. ((eye$ or ocular or vision) adj2 (care or health or service$)).tw.
5. visual acuity.tw.
6. (ophthalm$ or optomet$).tw.
7. exp eye disease/
8. (glaucoma$ or ocular hypertension or cataract$).tw.
9. ((diabet$ or proliferat$) adj3 retinopath$).tw.
10. (diabet$ adj3 (eye$ or vision or visual$ or sight$)).tw.
11. (retinopath$ adj3 (eye$ or vision or visual$ or sight$)).tw.
12. eye fundus/
13. (dilated adj2 fundus).tw.
14. (retinal adj2 exam$).tw.
15. (myop$ or hyperop$ or hypermetrop$ or anisometrop$ or ammetrop$ or astigmati$ or presbyop$).tw.
16. (refractive adj1 error$).tw.
17. spectacles/
18. (spectacle or spectacles or glasses).tw.
19. (eyeglasses or eye glasses).tw.
20. or/1-19
21. "ethnic or racial aspects"/
22. ethnic group/
23. minority group/
24. ((ethnic$ or racial$ or cultur$) adj3 (group$ or minorit$ or population$ or diverse$ or origin$)).tw.
25. ((ethnic$ or racial$ or cultur$) adj3 (inequalit$ or inequit$ or disparit$ or equit$ or disadvantage$ or depriv$)).tw.
26. ((population$ or communit$) adj3 (divers$ or disadvantage$ or depriv$)).tw.
27. non-Indigenous.tw.
28. (visible adj1 minorit$).tw.
29. refugee/
30. asylum seeker/
31. "emigrants and immigrants"/
32. (migrant$ or immigrant$ or emigrant$ or refugee$ or expatriate$).tw.
33. asylum seeker$.tw.
34. urban population/

For peer review only - http://bmjopen.bmj.com/site/about/guidelines.xhtml

35. urban poor.tw.
36. cultural factor/
37. cultural diversity/
38. cultural competence/
39. cultural deprivation/
40. ancestry group/
41. exp african american/
42. african caribbean/
43. black person/
44. negro/
45. "Caribbean (person)"/
46. asian continental ancestry group/
47. asian american/
48. population group/
49. hispanic/
50. geography.tw.
51. (Afr$ adj2 American$).tw.
52. (Afr$ adj2 Caribbean$).tw.
53. (west adj2 (india$ or indies)).tw.
54. (American$ adj1 black).tw.
55. (Asian$ or Indian$ or Pakistan$ or Bangladesh$ or Bengal$).tw.
56. ((Asia$ or Pacific) adj4 American$).tw.
57. ((Asia$ or Pacific) adj4 Islander$).tw.
58. (Hispanic or Latino or Latin American$ or Puerto Ric$ or Mexican$).tw.
59. or/21-58
60. program evaluation/
61. program$.tw.
62. ((educat$ or behaviour$ or behavior$ or improv$) adj3 (intervention$ or activat$)).tw.
63. health care delivery/
64. patient attitude/
65. health promotion/
66. (health adj2 (promotion$ or knowledge or belief$)).tw.
67. health education/
68. (educat$ adj2 (information or material or leaflet)).tw.
69. attitude to health/
70. health behavior/
71. patient education/
72. persuasive communication/
73. questionnaires/
74. information processing/
75. health survey/
76. health care survey/
77. exp interview/
78. (questionnaire$ or survey$).tw.
79. (focus adj3 group$).tw.
80. reminder system/
81. remind$.tw.
82. telephone/
83. telephone interview/
84. telephone.tw.
85. phone call.tw.
86. health behavior/
87. behavior therapy/
88. behavior change/
89. (behavioral or behavior or behaviour or behavioural).tw.
90. (increas$ adj3 (attend$ or uptake)).tw.
91. (approachability or acceptability or availability or affordability or appropriateness).tw.
92. (ability adj2 (perceive or seek or reach or pay or engage)).tw.
93. vision test/
94. mass screening/
95. (vision adj3 (test$ or screen$ or assess$)).tw.
96. (eye$ adj3 (test$ or screen$ or assess$)).tw.
97. ((target$ or tailor$) adj3 intervention$).tw.
98. (cultural$ adj3 (sensitiv$ or appropriate)).tw.
99. or/60-98
100. 20 and 59 and 99
101. exp developing country/
102. 100 not 101
103. prevalence.ti.
104. (genetic or mutation$ or autosomal or variant$).ti.
105. optical coherence tomography.tw.
106. (Latin American and Caribbean Health Sciences).tw.
107. or/103-106
108. 102 not 107
109. exp case report/
110. (case$ adj3 (report$ or series)).tw.
111. or/109-110
112. 108 not 111
113. limit 112 to conference abstract status
114. 112 not 113

For peer review only - http://bmjopen.bmj.com/site/about/guidelines.xhtml

Global Health (Ovid)

1. eyes/
2. eye diseases/
3. vision/
4. vision disorders/
5. ((eye$ or ocular or vision) adj2 (care or health or service$)).tw.
6. visual acuity.tw.
7. (ophthalm$ or optomet$).tw.
8. (glaucoma$ or ocular hypertension or cataract$).tw.
9. ((diabet$ or proliferat$) adj3 retinopath$).tw.
10. (diabet$ adj3 (eye$ or vision or visual$ or sight$)).tw.
11. (retinopath$ adj3 (eye$ or vision or visual$ or sight$)).tw.
12. (dilated adj2 fundus).tw.
13. (retinal adj2 exam$).tw.
14. (myop$ or hyperop$ or hypermetrop$ or anisometrop$ or ammetrop$ or astigmati$ or presbyop$).tw.
15. (refractive adj1 error$).tw.
16. (spectacle or spectacles or glasses).tw.
17. (eyeglasses or eye glasses).tw.
18. or/1-17
19. ethnic groups/
20. ethnicity/
21. minorities/
22. ((ethnic$ or racial$ or cultur$) adj3 (group$ or minorit$ or population$ or diverse$ or origin$)).tw.
23. ((ethnic$ or racial$ or cultur$) adj3 (inequalit$ or inequit$ or disparit$ or equit$ or disadvantage$ or depriv$)).tw.
24. ((population$ or communit$) adj3 (divers$ or disadvantage$ or depriv$)).tw.
25. non-Indigenous.tw.
26. (visible adj1 minorit$).tw.
27. refugees/
28. immigrants/
29. immigration/
30. (migrant$ or immigrant$ or emigrant$ or refugee$ or expatriate$).tw.
31. asylum seeker$.tw.
32. urban population/
33. urban areas/
34. urban poor.tw.
35. geography.tw.
36. african americans/ or african-caribbeans/ or american indians/ or asians/ or black people/ or japanese americans/ or mexican-americans/ or pacific islanders/
37. (Afr$ adj2 American$).tw.
38. (Afr$ adj2 Caribbean$).tw.
39. (west adj2 (india$ or indies)).tw.
40. (American$ adj1 black).tw.
41. (Asian$ or Indian$ or Pakistan$ or Bangladesh$ or Bengal$).tw.
42. ((Asia$ or Pacific) adj4 American$).tw.
43. or/19-42
44. program evaluation/
45. programs/
46. program$.tw.
47. ((educat$ or behaviour$ or behavior$ or improv$) adj3 (intervention$ or activat$)).tw.
48. (primary health care or health services or community health or health policy or health care or public health or health programmes).sh.
49. health promotion/
50. (health adj2 (promotion$ or knowledge or belief$)).tw.
51. health education/
52. patient education/
53. (educat$ adj2 (information or material or leaflet)).tw.
54. attitude/
55. communication/
56. questionnaires/
57. discussion groups/
58. surveys/
59. (questionnaire$ or survey$).tw.
60. (focus adj3 group$).tw.
61. remind$.tw.
62. telephone/
63. telephone.tw.
64. phone call.tw.

For peer review only - http://bmjopen.bmj.com/site/about/guidelines.xhtml

65. behaviour/
66. (behavioral or behavior or behaviour or behavioural).tw.
67. (increas$ adj3 (attend$ or uptake)).tw.
68. (approachability or acceptability or availability or affordability or appropriateness).tw.
69. (ability adj2 (perceive or seek or reach or pay or engage)).tw.
70. screening/
71. (vision adj3 (test$ or screen$ or assess$)).tw.
72. (eye$ adj3 (test$ or screen$ or assess$)).tw.
73. ((target$ or tailor$) adj3 intervention$).tw.
74. (cultural$ adj3 (sensitiv$ or appropriate)).tw.
75. culture/
76. or/44-75
77. 18 and 43 and 76
78. exp developing countries/
79. 77 not 78
80. prevalence.ti.
81. (genetic or mutation$ or autosomal or variant$).ti.
82. optical coherence tomography.tw.
83. (Latin American and Caribbean Health Sciences).tw.
84. or/80-83
85. 79 not 84
86. exp case report/
87. (case$ adj3 (report$ or series)).tw.
88. 86 or 87
89. 85 not 88

For peer review only

For peer review only - http://bmjopen.bmj.com/site/about/guidelines.xhtml

BMJ Open

Interventions to promote access to eye care for non-Indigenous, non-dominant ethnic groups in high-income countries: a scoping review protocol

Journal:	*BMJ Open*
Manuscript ID	bmjopen-2019-033775.R2
Article Type:	Protocol
Date Submitted by the Author:	29-Apr-2020
Complete List of Authors:	Hamm, Lisa; The University of Auckland, School of Optometry and Vision Science<br>Black, Joanna; The University of Auckland, School of Optometry and Vision Science<br>Burn, Helen; Brighton and Sussex University Hospitals NHS Trust<br>Grey, Corina; University of Auckland, School of Population Health<br>Harwood, Matire; University of Auckland, School of Population Health<br>Peiris-John, Roshini; The University of Auckland, School of Population Health<br>Gordon, Iris; London School of Hygiene and Tropical Medicine Faculty of Infectious and Tropical Diseases, International Centre for Eye Health<br>Burton, Matthew J; London School of Hygiene and Tropical Medicine<br>Evans, Jennifer; London School of Hygiene and Tropical Medicine Faculty of Infectious and Tropical Diseases, International Centre for Eye Health<br>Ramke, Jacqueline; London School of Hygiene and Tropical Medicine Faculty of Infectious and Tropical Diseases, International Centre for Eye Health; The University of Auckland, School of Optometry and Vision Science
**Primary Subject Heading**:	Ophthalmology
Secondary Subject Heading:	Global health, Health services research, Patient-centred medicine
Keywords:	ethnic minority, ethnic disparity, immigrants, eye care, optometry

SCHOLARONE™
Manuscripts

BMJ

I, the Submitting Author has the right to grant and does grant on behalf of all authors of the Work (as defined in the below author licence), an exclusive licence and/or a non-exclusive licence for contributions from authors who are: i) UK Crown employees; ii) where BMJ has agreed a CC-BY licence shall apply, and/or iii) in accordance with the terms applicable for US Federal Government officers or employees acting as part of their official duties; on a worldwide, perpetual, irrevocable, royalty-free basis to BMJ Publishing Group Ltd ("BMJ") its licensees and where the relevant Journal is co-owned by BMJ to the co-owners of the Journal, to publish the Work in this journal and any other BMJ products and to exploit all rights, as set out in our licence.

The Submitting Author accepts and understands that any supply made under these terms is made by BMJ to the Submitting Author unless you are acting as an employee on behalf of your employer or a postgraduate student of an affiliated institution which is paying any applicable article publishing charge ("APC") for Open Access articles. Where the Submitting Author wishes to make the Work available on an Open Access basis (and intends to pay the relevant APC), the terms of reuse of such Open Access shall be governed by a Creative Commons licence – details of these licences and which Creative Commons licence will apply to this Work are set out in our licence referred to above.

Other than as permitted in any relevant BMJ Author's Self Archiving Policies, I confirm this Work has not been accepted for publication elsewhere, is not being considered for publication elsewhere and does not duplicate material already published. I confirm all authors consent to publication of this Work and authorise the granting of this licence.

For peer review only

Interventions to promote access to eye care for non-Indigenous, non-dominant ethnic groups in high-income countries: a scoping review protocol

Lisa M **Hamm**[1]	l.hamm@auckland.ac.nz	0000-0003-2777-7146
Joanna **Black**[1]	j.black@auckland.ac.nz	0000-0002-5100-8796
Helen **Burn**[2]	hburn88@gmail.com	0000-0002-1469-8169
Corina **Grey**[3]	c.grey@auckland.ac.nz	0000-0003-1751-1934
Matire **Harwood**[3]	m.harwood@auckland.ac.nz	0000-0003-1240-5139
Roshini **Peiris-John**[3]	r.peiris-john@auckland.ac.nz	0000-0001-7812-2268
Iris **Gordon**[2]	Iris.Gordon@lshtm.ac.uk	0000-0001-8143-8132
Matthew J **Burton**[2,4]	Matthew.Burton@lshtm.ac.uk	0000-0003-1872-9169
Jennifer R **Evans**[2]	Jennifer.Evans@lshtm.ac.uk	0000-0002-6137-2030
Jacqueline **Ramke**[1,2]	Jacqueline.Ramke@lshtm.ac.uk	0000-0002-5764-1306

1. School of Optometry & Vision Science, Faculty of Medicine and Health Sciences, The University of Auckland, Auckland, New Zealand
2. International Centre for Eye Health, London School of Hygiene and Tropical Medicine, London, United Kingdom
3. School of Population Health, Faculty of Medicine and Health Sciences, University of Auckland, Auckland, New Zealand
4. Moorfields Eye Hospital, London, United Kingdom

Author Contributors: JR conceived the idea for the review. LMH and JR drafted and revised the protocol with suggestions from JB, HB, CG, MH, RPJ, MJB and JE who reviewed the protocol and provided feedback on the draft. IG constructed the search.

Corresponding Author: Jacqueline Ramke (jacqueline.ramke@lshtm.ac.uk)

Funding Statement: This work was supported by The University of Auckland Faculty Research Development Fund grant number 3716758. JR is a Commonwealth Rutherford Fellow, funded by the UK government through the Commonwealth Scholarship Commission in the UK. LMH is the Robert Leitl Research Fellow, funded by the Robert Leitl Trust and the University of Auckland School of Optometry and Vision Science. CG holds a National Heart Foundation Fellowship. MJB is supported by the Wellcome Trust (207472/Z/17/Z).

Competing Interests: None declared. **Table Number**: 0. **Figure Number**: 1

Patient and Public Involvement Statement: As this was a review of existing literature, this research was done without specific patient involvement.

Data Sharing Statement: Data generated from this review will be available upon reasonable request from Jacqueline Ramke (jacqueline.ramke@lshtm.ac.uk)

Keywords: non-dominant ethnic group, ethnic minority, ethnic disparity, immigrants, eye care, optometry, ophthalmology, access, service delivery, health

Word count: 2240

For peer review only - http://bmjopen.bmj.com/site/about/guidelines.xhtml

ABSTRACT

Introduction

For many people, settling in a new country is associated with a new identity as an 'ethnic minority', one that can remain through future generations. People who are culturally distinct from the dominant population group may experience a variety of barriers to accessing health care, including linguistic and cultural barriers in communication, navigation of an unfamiliar health system, and unconscious or overt discrimination. Here we outline the protocol of a scoping review to identify, describe and summarise interventions aimed at improving access to eye care for non-Indigenous, non-dominant ethnic groups residing in high-income countries.

Methods and analysis

We will search MEDLINE, Embase and Global Health from their inception to July 2019. We will include studies of any design that describe an intervention to promote access to eye care for non-Indigenous, non-dominant ethnic groups. Two authors will independently review titles, abstracts, and full-text articles for inclusion. Reference lists from all included articles will also be searched. In cases of disagreement between initial reviewers, a third author will help resolve the conflict. For each included article we will extract data about the target population, details of the intervention delivered and the effectiveness of or feedback from the intervention. Overall findings will be summarised with descriptive statistics and thematic analysis.

Ethics and dissemination

This review will summarise existing literature and as such ethics approval is not required. We will publish the review in an open-access, peer-reviewed journal, and draft appropriate summaries for dissemination to the wider community. This wider community could include clinicians, policymakers, health service managers and organisations that work with vulnerable ethnic minorities. Our findings will also feed into the ongoing *Lancet Global Health* Commission on Global Eye Health.

For peer review only

For peer review only - http://bmjopen.bmj.com/site/about/guidelines.xhtml

Strengths and limitations of this study

- This study will provide a comprehensive overview of the published literature on interventions to improve access to eye care for non-Indigenous, non-dominant ethnic groups in high-income countries.
- The review will be comprehensive, including published literature of all study designs, without time period or language restrictions.
- A potential limitation is that the population of interest can be difficult to define.
- Relevant evidence may exist in the grey literature, but our review is limited to the published literature.

For peer review only

For peer review only - http://bmjopen.bmj.com/site/about/guidelines.xhtml

INTRODUCTION

Rationale

Equitable access to health care is critically important, but it is a challenge to both define and achieve.[1] Health systems are often implicitly structured to meet the needs and preferences of members of the dominant group in any given population, which makes these systems more challenging for people with diverse backgrounds to navigate.[2 3] The axes of diversity vary widely (including socio-economic status, gender, sexual orientation, Indigeneity) and are often intersectional. Challenges in navigation of health care systems are compounded for people with a non-dominant ethnic background, because the health care seeker is more likely to look, speak, and communicate differently to their health care providers.[4]

The history of each ethnic group in a given place can influence how and to what extent health services strive to mitigate the vulnerabilities experienced by the group. For example, there is increasing recognition of overt institutional racism against colonised Indigenous populations and the impact this has on health care and health outcomes.[5] Formal efforts at restitution[6] have attempted to improve access to health care such as government-funded services to rural and remote areas with high Indigenous populations, and health facilities within Indigenous communities. For this reason, we are investigating service delivery models to improve access to eye care for Indigenous populations in a complementary scoping review,[7] and in the scoping review outlined here, we consider interventions to promote access to eye care for *non-Indigenous*, non-dominant ethnic groups.

A 'migrant' is a person who is living or has lived in a different place than they were born.[3] Using this definition, it was estimated that 3.4% of the global population (258 million people) were migrants in 2017.[8] People move away from their country of birth for a variety of reasons; many move for employment, others are forced from their home country because of civil unrest or violence, and others are moved through human trafficking and modern slavery.[3] Some migrants arrive in countries with a similar culture and language to their own, while others are faced with navigating a new cultural context, often finding themselves misunderstood or discriminated against, and subject to many barriers to accessing quality health care.[3 4] These challenges can endure through future generations, with many people treated like perpetual foreigners in the only home they have known.

Non-dominant ethnic groups are vulnerable to poor access to health care in several ways. A lack of familiarity with local health systems or a fear that using community resources might compromise social acceptance or immigration status can prevent people from seeking care.[9] When people from non-dominant ethnic groups do seek care, the health care provider is unlikely to share their native language or cultural heritage.[10] This can be associated with unarticulated differences in cultural beliefs about health[11] and a general breakdown in rapport or trust.[12] In the worst case, people are overtly disrespected in medical environments, compromising future health seeking behaviour.[10] Breakdowns in understanding are often magnified at a structural level. People from non-dominant

For peer review only

For peer review only - http://bmjopen.bmj.com/site/about/guidelines.xhtml

ethnic groups often have limited power to impact the systems around them; they are less likely to be included in decision-making structures, or to be identified as a priority group for health funding.[3]

Similar issues impact eye care.[13 14] Studies from the USA report underutilization of eye care services by non-dominant ethnic groups in general[15] and specifically by Latin Americans[16] and recent immigrants.[17] Although some public services are available (e.g. Medicaid includes eye care and some school vision screening programs) the ability to fully use services is often compromised.[13 18 19]

Some interventions exist to promote access to quality eye care for vulnerable ethnic groups[17 20 21] however these studies are diverse in terms of the population targeted, the methodological framework, and the eye problem addressed. Indeed, defining the target group is a challenge, given the difficulty in defining ethnicity and the overlap of ethnicity with socioeconomic status, education, acculturation and geography. Given this diversity, a systematic review may be inconclusive at this time, and further primary studies would not adequately build on lessons learned within this literature. A scoping review[22] appeared the most appropriate method to map and summarise this field of research.[23] This protocol (and a parallel protocol[7]) were designed to inform a project to improve access to eye care services for Māori and Pacific people in Aotearoa New Zealand, but the scope and implications are international.

Definitions and concepts

The group of primary interest in this review is difficult to define. Self-identification of ethnicity is often fluid and nuanced,[24] and appropriate terminology within health research is actively debated.[25] Although 'ethnic minority' is commonly used in health research to refer to a group with a shared ethnic or cultural heritage which differs from the dominant population where one resides, there is no accepted international definition.[6] For example, 'minority' can mean numerically smaller, or it can reference lack of power or dominance.[6] Some definitions include a will to preserve a cultural identity, while others note that group membership is involuntary or imposed (this distinction is sometimes captured in the differential use of 'ethnicity' as self-identity vs 'race' as an imposed identity[25]). Indeed, many terms related to the role of ethnicity in society carry different implicit meaning across countries and time. For example, terms like 'migrant', 'immigrant' and 'expatriate' each reflect a new identity when living in a new place, yet differential use can reveal assumptions about perceived wealth and influence. Similarly, the terms 'race', 'ethnicity', 'national' and 'visible minority' can carry nuanced assumptions that may not be shared internationally.

Within these challenges of terminology, we are interested in ethnic identities which are disempowering in their immediate context, and we refer to these groups as 'non-dominant ethnic groups'. This could include refugees and recent immigrants as well as those who have lived in the country of residence for many generations. Since we have chosen to address Indigenous populations in a separate review,[7] our definition here is limited to people who are not Indigenous to the country in which the study is located.

For peer review only - http://bmjopen.bmj.com/site/about/guidelines.xhtml

We have defined *eye care service delivery intervention* as any organised programme or activity designed to improve access to care, according to the patient-centred access to health care framework provided by Levesque et al.[26] Levesque's framework includes a progression from health care needs to perception of needs and desire for care, to health care seeking, reaching, utilisation, and finally consequences (Figure 1). The progression between stages depends on services factors, including acceptability, accessibility, availability and accommodation, affordability, and appropriateness, as well as the resources and knowledge of the patient, including their ability to perceive, seek, reach, pay for and engage with health care services.

The *eye care* that will be covered will include general services (prevention and treatment services, as well as vision rehabilitation), as well as those for a particular condition or age group.

Insert Figure 1 here

Figure 1: Conceptual framework for access to healthcare (reproduced from Levesque *et al.* [26]*)*

METHODS AND ANALYSIS

We have reported this protocol in accordance with the relevant sections of the PRISMA-ScR guideline.[27] The same guideline will be used to report the final review.

Scoping review questions

We aim to answer three key questions:

1) What is the extent of the published literature on interventions to promote access to eye care for non-Indigenous non-dominant ethnic groups living in high-income countries?

2) What can we learn from reported effectiveness of interventions?

3) What can we learn from authors' reflections on the potential to improve upon the interventions?

Eligibility criteria

We are interested in studies that describe interventions to improve access to eye care services for non-dominant ethnic groups (as defined above) residing in high-income countries (as defined by the World Bank[28]). This includes recent migrants, refugees and those who have resided in the high-income country for multiple generations. Given the interplay between ethnicity and socio-economic status,[29] interventions which could improve eye care services for ethnic minorities may have targeted another population (for example 'urban poor'), but had a high proportion of participants from non-dominant ethnic groups. When these studies inform our objectives, we will include studies in which at least 50% of participants are from any non-dominant, non-Indigenous population group. Given the exploratory nature of a scoping review, we will iteratively discuss this component of the inclusion criteria with an aim to include the most relevant papers and will note any changes in the final review.

For peer review only - http://bmjopen.bmj.com/site/about/guidelines.xhtml

For peer review only

Studies which aim to improve provision of health care more generally may include eye care e.g. interventions for diabetes care may include an assessment of diabetic retinopathy. In these cases, we will only include studies if there is sufficient detail on the eye care component of the intervention to be relevant as a standalone resource. We will exclude reviews, commentaries and editorials, but will check the reference list of review articles for potentially relevant studies. We will include all languages and study designs but will exclude studies for which the full text is unavailable after exhausting university library resources.

Search strategy

The authors collaborated to propose relevant search terms. Final terms and strategy (details in Supplementary File 1) were then refined for use within MEDLINE, Embase and Global Health databases by Cochrane Eyes and Vision's Information Specialist (IG). Literature will be searched to July 2019. An additional round of searching will be based on reference lists from included articles and relevant reviews. Due to resource constraints we will not search grey literature.

Study selection

All the results from the search will be entered into Covidence (www.covidence.org) for screening. Two authors (from LMH, JR, JB, CG, RPJ or HB) will independently review each title and abstract and exclude those that do not meet the inclusion criteria. If the reviewers do not agree, the two reviewers will discuss and resolve. A third author will be consulted if no resolution can be found by the initial two reviewers. The full text of the selected articles will be reviewed, and the same two authors will independently vote to include or exclude articles. Again, conflict resolution will be handled by discussion, and a third reviewer if needed. A PRISMA flow diagram will be used to summarise the screening process.

Data charting

A data extraction form will be developed based on the data items detailed below. The form will be piloted on five studies by each of LMH, JR, JB, CG and HB, and amendments made. Given the diversity of expected results, the charting process will remain iterative, with all changes to the data charting process noted. As with the screening process, two authors will independently chart each included article. Differences in charting will be resolved through discussion, and a third author called on to resolve discrepancies if needed. If additional information is required from included studies, we will contact authors directly via email with a maximum of three attempts.

Data items

The following data items will be collected during the data charting process:

1. Publication characteristics: (title, year of publication, study design, country of origin, study setting)
2. Characteristics of the targeted group(s): (age, ethnicity, language, socioeconomic status, duration of residence in place of study)

For peer review only - http://bmjopen.bmj.com/site/about/guidelines.xhtml

3. Characteristics of the intervention (eye care context, targeted population's involvement in the development and implementation of the intervention, what was done to improve access to eye health, which dimensions of the Levesque framework for access were addressed and how).
4. Evaluated outcomes of the study (if the intervention was evaluated, how was it evaluated and what was the effectiveness, how many people were impacted by the intervention, how were baseline values and outcomes measured, and what analyses were used to draw conclusions)
5. Authors' reflections on the intervention (Authors' reflections on what worked and why, what did not and why, and any suggestions for future interventions).

Data synthesis

The interventions will be summarised descriptively and grouped according to the context, target population, eye condition(s) and access dimensions outlined above. Where interventions have been evaluated, the effectiveness, as well as identified strengths, weaknesses and suggested future directions will be summarised.

ETHICS AND DISSEMINATION

This review will summarise existing literature and as such ethics approval is not required. We will publish the review in an open-access, peer-reviewed journal, and draft appropriate summaries for dissemination to the wider community. This wider community could include clinicians, policymakers, health service managers and organisations that work with vulnerable ethnic minorities. Our findings will also feed into the ongoing *Lancet Global Health* Commission on Global Eye Health.[30]

DISCUSSION

The challenges faced by some migrants in a new country can persist through many generations.[6] Access to public services, including eye care, is one such challenge.[14] The barriers are varied, influenced both by health system structures, leaders and workforce, and the resources and knowledge of the patient.[26] Given the diverse communities, with diverse barriers to eye care, varied interventions to improve access to eye care are likely to be needed. Here we have outlined a protocol for a scoping review to summarise interventions to improve access to eye care for people from non-dominant ethnic groups.

We aim to map the available literature on the topic, which may take many forms. A scoping review lends itself well to this endeavour, especially given the anticipated diversity of the work in the field.[22] The scoping review outlined here is part of a larger study to improve access to eye care services for Indigenous and non-Indigenous ethnic groups in Aotearoa New Zealand. The findings will be useful to policymakers, health service managers and clinicians responsible for eye care services in New Zealand, as well as in other countries with similar marginalised population groups. In addition to publication in an open access journal, we will develop an accessible summary of the results for posting on institutional websites and dissemination at stakeholder meetings.

For peer review only - http://bmjopen.bmj.com/site/about/guidelines.xhtml

For peer review only

REFERENCES

1. Braveman P. Health disparities and health equity: Concepts and measurement. *Annual Review of Public Health*, 2006:167-94.
2. Dawson AJ, Nkowane AM, Whelan A. Approaches to improving the contribution of the nursing and midwifery workforce to increasing universal access to primary health care for vulnerable populations: a systematic review. *Hum Resour Health* 2015;13:97. doi: 10.1186/s12960-015-0096-1
3. Abubakar I, Aldridge RW, Devakumar D, et al. The UCL-Lancet Commission on Migration and Health: the health of a world on the move. *Lancet* 2018;392(10164):2606-54. doi: 10.1016/S0140-6736(18)32114-7
4. Cooper LA, Hill MN, Powe NR. Designing and evaluating interventions to eliminate racial and ethnic disparities in health care. *J Gen Intern Med* 2002;17(6):477-86.
5. Anderson I, Robson B, Connolly M, et al. Indigenous and tribal peoples' health (The Lancet–Lowitja Institute Global Collaboration): a population study. *The Lancet* 2016;388(10040):131-57. doi: 10.1016/S0140-6736(16)00345-7
6. Indigenous peoples and ethnic minorities: Marginalization is the norm. Promoting inclusion through social protection United Nations 2018.
7. Burn H, Black J, Harwood M, et al. Eye care delivery models to improve access to eye care for Indigenous peoples in high income countries: protocol for a scoping review *BMJ Open* 2019, in press
8. Chokron S, Cavezian C, Gaudry I. Cortical visual impairment in children and their relationship with learning disorders. *ANAE - Approche Neuropsychologique des Apprentissages chez l'Enfant* 2014;26(129):117-26.
9. Hall AG. Medicaid's impact on access to and utilization of health care services among racial and ethnic minority children. *J Urban Health* 1998;75(4):677-92. doi: 10.1007/BF02344498
10. Rutledge EO. The struggle for equality in healthcare continues. *J Healthc Manag* 2001;46(5):313-24; discussion 24-6.
11. Wilson D, Heaslip V, Jackson D. Improving equity and cultural responsiveness with marginalised communities: Understanding competing worldviews. *J Clin Nurs* 2018;27(19-20):3810-19. doi: 10.1111/jocn.14546
12. Boulware LE, Cooper LA, Ratner LE, et al. Race and trust in the health care system. *Public health reports (Washington, DC : 1974)* 2003;118(4):358-65. doi: 10.1093/phr/118.4.358 [published Online First: 2003/06/20]
13. Islam NS, Kwon SC, Wyatt LC, et al. Disparities in diabetes management in Asian Americans in New York City compared with other racial/ethnic minority groups. *American Journal of Public Health* 2015;105:S443-S46. doi: 10.2105/AJPH.2014.302523
14. Varadaraj V, Frick KD, Saaddine JB, et al. Trends in Eye Care Use and Eyeglasses Affordability: The US National Health Interview Survey, 2008-2016Trends in Eye Care Use and Eyeglasses AffordabilityTrends in Eye Care Use and Eyeglasses Affordability. *JAMA Ophthalmology* 2019;137(4):391-98. doi: 10.1001/jamaophthalmol.2018.6799
15. Baker RS, Bazargan M, Bazargan-Hejazi S, et al. Access to vision care in an urban low-income multiethnic population. *Ophthalmic Epidemiol* 2005;12(1):1-12. doi: 10.1080/09286580590921330
16. Varma R, Mohanty SA, Deneen J, et al. Burden and predictors of undetected eye disease in Mexican-Americans: the Los Angeles Latino Eye Study. *Med Care* 2008;46(5):497-506. doi: 10.1097/MLR.0b013e31816080fe
17. Martin SA, Frutiger EA. Vision Stations: Addressing Corrective Vision Needs With Low-cost Technologies. *Glob Adv Health Med* 2015;4(2):46-51. doi: 10.7453/gahmj.2015.002
18. Basch CE. Vision and the achievement gap among urban minority youth. *J Sch Health* 2011;81(10):599-605. doi: 10.1111/j.1746-1561.2011.00633.x
19. Lee B. Reducing Disparities in Pediatric Vision Care Utilization by Strategically Addressing Access. *JAMA Ophthalmol* 2018 doi: 10.1001/jamaophthalmol.2018.5939
20. Walker EA, Schechter CB, Caban A, et al. Telephone intervention to promote diabetic retinopathy screening among the urban poor. *Am J Prev Med* 2008;34(3):185-91. doi: 10.1016/j.amepre.2007.11.020
21. Jones HL, Walker EA, Schechter CB, et al. Vision is precious: a successful behavioral intervention to increase the rate of screening for diabetic retinopathy for inner-city adults. *Diabetes Educ* 2010;36(1):118-26. doi: 10.1177/0145721709356116
22. Arksey H, O'Malley L. Scoping studies: towards a methodological framework. *International Journal of Social Research Methodology* 2005;8(1):19-32. doi: 10.1080/1364557032000119616

For peer review only - http://bmjopen.bmj.com/site/about/guidelines.xhtml

23. Munn Z, Peters MDJ, Stern C, et al. Systematic review or scoping review? Guidance for authors when choosing between a systematic or scoping review approach. *BMC Medical Research Methodology* 2018;18(1):143. doi: 10.1186/s12874-018-0611-x
24. Merino ME, Tileagă C. The construction of ethnic minority identity: A discursive psychological approach to ethnic self-definition in action. *Discourse and Society* 2011;22(1):86-101. doi: 10.1177/0957926510382834
25. Ford CL, Harawa NT. A new conceptualization of ethnicity for social epidemiologic and health equity research. *Social Science and Medicine* 2010;71(2):251-58. doi: 10.1016/j.socscimed.2010.04.008
26. Levesque JF, Harris MF, Russell G. Patient-centred access to health care: conceptualising access at the interface of health systems and populations. *Int J Equity Health* 2013;12:18. doi: 10.1186/1475-9276-12-18
27. Tricco AC, Lillie E, Zarin W, et al. PRISMA Extension for Scoping Reviews (PRISMA-ScR): Checklist and Explanation. *Ann Intern Med* 2018;169(7):467-73. doi: 10.7326/M18-0850
28. Watson T, Orel-Bixler D, Haegerstrom-Portnoy G. Early visual-evoked potential acuity and future behavioral acuity in cortical visual impairment. *Optometry and Vision Science* 2010;87(2):80-86. doi: 10.1097/OPX.0b013e3181c75184
29. Kawachi I, Daniels N, Robinson DER. Health Disparities By Race And Class: Why Both Matter. *Health Affairs* 2005;24(2):343-52. doi: 10.1377/hlthaff.24.2.343
30. Burton MJ, Faal HB, Ramke J, et al. Announcing The Lancet Global Health Commission on Global Eye Health. *The Lancet Global Health* 2019;7(12):e1612-e13. doi: 10.1016/S2214-109X(19)30450-4

For peer review only

For peer review only - http://bmjopen.bmj.com/site/about/guidelines.xhtml

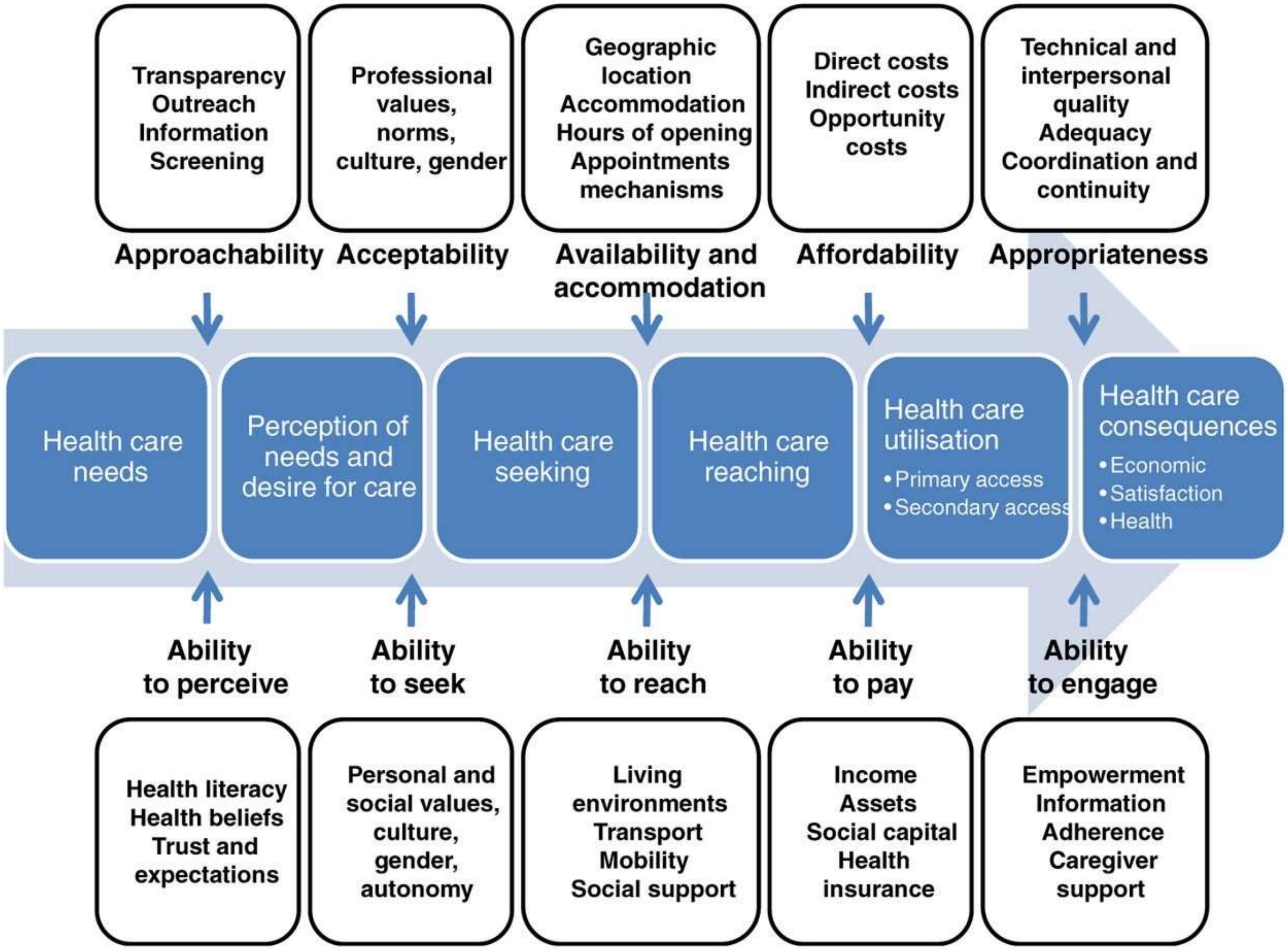
Transparency
Outreach
Information
Screening
Professional values, norms, culture, gender
Geographic location
Accommodation
Hours of opening
Appointments mechanisms
Direct costs
Indirect costs
Opportunity costs
Technical and interpersonal quality
Adequacy
Coordination and continuity
Approachability
Acceptability
Availability and accommodation
Affordability
Appropriateness
Health care needs
Perception of needs and desire for care
Health care seeking
Health care reaching
Health care utilisation
• Primary access
• Secondary access
Health care consequences
• Economic
• Satisfaction
• Health
Ability to perceive
Ability to seek
Ability to reach
Ability to pay
Ability to engage
Health literacy
Health beliefs
Trust and expectations
Personal and social values, culture, gender, autonomy
Living environments
Transport
Mobility
Social support
Income
Assets
Social capital
Health insurance
Empowerment
Information
Adherence
Caregiver support

Appendix: Search strategy

MEDLINE (Ovid)

1. exp Ophthalmology/
2. Optometry/
3. ((eye$ or ocular or vision) adj2 (care or health or service$)).tw.
4. visual acuity.tw.
5. (ophthalm$ or optomet$).tw.
6. exp Eye Diseases/
7. (glaucoma$ or ocular hypertension or cataract$).tw.
8. ((diabet$ or proliferat$) adj3 retinopath$).tw.
9. (diabet$ adj3 (eye$ or vision or visual$ or sight$)).tw.
10. (retinopath$ adj3 (eye$ or vision or visual$ or sight$)).tw.
11. (dilated adj2 fundus).tw.
12. (retinal adj2 exam$).tw.
13. (myop$ or hyperop$ or hypermetrop$ or anisometrop$ or ammetrop$ or astigmati$ or presbyop$).tw.
14. (refractive adj1 error$).tw.
15. Eyeglasses/
16. (spectacle or spectacles or glasses).tw.
17. (eyeglasses or eye glasses).tw.
18. or/1-17
19. Ethnic Groups/
20. Minority Groups/
21. ((ethnic$ or racial$ or cultur$) adj3 (group$ or minorit$ or population$ or diverse$ or origin$)).tw.
22. ((ethnic$ or racial$ or cultur$) adj3 (inequalit$ or inequit$ or disparit$ or equit$ or disadvantage$ or depriv$)).tw.
23. ((population$ or communit$) adj3 (divers$ or disadvantage$ or depriv$)).tw.
24. non-Indigenous.tw.
25. (visible adj1 minorit$).tw.
26. Refugees/
27. "emigrants and immigrants"/
28. (migrant$ or immigrant$ or emigrant$ or refugee$ or expatriate$).tw.
29. asylum seeker$.tw.
30. Urban Population/
31. urban poor.tw.
32. Cultural Characteristics/
33. Cross-Cultural Comparison/
34. Cultural Diversity/
35. Cultural competency/
36. Cultural deprivation/
37. exp African Continental Ancestry Group/
38. exp Asian Continental Ancestry Group/
39. Continental Population Groups/
40. geography.tw.
41. (Afr$ adj2 American$).tw.
42. (Afr$ adj2 Caribbean$).tw.
43. (west adj2 (india$ or indies)).tw.
44. (American$ adj1 black).tw.
45. (Asian$ or Indian$ or Pakistan$ or Bangladesh$ or Bengal$).tw.
46. ((Asia$ or Pacific) adj4 American$).tw.
47. ((Asia$ or Pacific) adj4 Islander$).tw.
48. (Hispanic or Latino or Latin American$ or Puerto Ric$ or Mexican$).tw.
49. or/19-48
50. Program Evaluation/
51. program$.tw.
52. ((educat$ or behaviour$ or behavior$ or improv$) adj3 (intervention$ or activat$)).tw.
53. Delivery of Health Care/
54. Health Services Accessibility/
55. Patient Acceptance of Health Care/
56. Health Promotion/
57. (health adj2 (promotion$ or knowledge or belief$)).tw.
58. Health Education/
59. (educat$ adj2 (information or material or leaflet)).tw.
60. Health Knowledge, Attitudes, Practice/
61. Patient Education as Topic/
62. Persuasive Communication/
63. "Surveys and Questionnaires"/
64. Questionnaires/
65. Focus Groups/
66. Health Surveys/
67. Health Care Surveys/

For peer review only - http://bmjopen.bmj.com/site/about/guidelines.xhtml

68. Interviews as Topic/
69. (questionnaire$ or survey$).tw.
70. (focus adj3 group$).tw.
71. exp Reminder Systems/
72. remind$.tw.
73. Telephone/
74. telephone.tw.
75. phone call.tw.
76. Health Behavior/
77. Behavior Therapy/
78. (behavioral or behavior or behaviour or behavioural).tw.
79. (increas$ adj3 (attend$ or uptake)).tw.
80. (approachability or acceptability or availability or affordability or appropriateness).tw.
81. (ability adj2 (perceive or seek or reach or pay or engage)).tw.
82. exp Vision Tests/
83. Mass Screening/
84. (vision adj3 (test$ or screen$ or assess$)).tw.
85. (eye$ adj3 (test$ or screen$ or assess$)).tw.
86. ((target$ or tailor$) adj3 intervention$).tw.
87. (cultural$ adj3 (sensitiv$ or appropriate)).tw.
88. or/50-87
89. 18 and 49 and 88
90. exp developing countries/
91. 89 not 90
92. prevalence.ti.
93. (genetic or mutation$ or autosomal or variant$).ti.
94. optical coherence tomography.tw.
95. (Latin American and Caribbean Health Sciences).tw.
96. or/92-95
97. 91 not 96
98. exp case report/
99. (case$ adj3 (report$ or series)).tw.
100. 98 or 99
101. 97 not 100

Embase (Ovid)

1. ophthalmology/
2. optometry/
3. optometrist/
4. ((eye$ or ocular or vision) adj2 (care or health or service$)).tw.
5. visual acuity.tw.
6. (ophthalm$ or optomet$).tw.
7. exp eye disease/
8. (glaucoma$ or ocular hypertension or cataract$).tw.
9. ((diabet$ or proliferat$) adj3 retinopath$).tw.
10. (diabet$ adj3 (eye$ or vision or visual$ or sight$)).tw.
11. (retinopath$ adj3 (eye$ or vision or visual$ or sight$)).tw.
12. eye fundus/
13. (dilated adj2 fundus).tw.
14. (retinal adj2 exam$).tw.
15. (myop$ or hyperop$ or hypermetrop$ or anisometrop$ or ammetrop$ or astigmati$ or presbyop$).tw.
16. (refractive adj1 error$).tw.
17. spectacles/
18. (spectacle or spectacles or glasses).tw.
19. (eyeglasses or eye glasses).tw.
20. or/1-19
21. "ethnic or racial aspects"/
22. ethnic group/
23. minority group/
24. ((ethnic$ or racial$ or cultur$) adj3 (group$ or minorit$ or population$ or diverse$ or origin$)).tw.
25. ((ethnic$ or racial$ or cultur$) adj3 (inequalit$ or inequit$ or disparit$ or equit$ or disadvantage$ or depriv$)).tw.
26. ((population$ or communit$) adj3 (divers$ or disadvantage$ or depriv$)).tw.
27. non-Indigenous.tw.
28. (visible adj1 minorit$).tw.
29. refugee/
30. asylum seeker/
31. "emigrants and immigrants"/
32. (migrant$ or immigrant$ or emigrant$ or refugee$ or expatriate$).tw.
33. asylum seeker$.tw.
34. urban population/

For peer review only - http://bmjopen.bmj.com/site/about/guidelines.xhtml

35. urban poor.tw.
36. cultural factor/
37. cultural diversity/
38. cultural competence/
39. cultural deprivation/
40. ancestry group/
41. exp african american/
42. african caribbean/
43. black person/
44. negro/
45. "Caribbean (person)"/
46. asian continental ancestry group/
47. asian american/
48. population group/
49. hispanic/
50. geography.tw.
51. (Afr$ adj2 American$).tw.
52. (Afr$ adj2 Caribbean$).tw.
53. (west adj2 (india$ or indies)).tw.
54. (American$ adj1 black).tw.
55. (Asian$ or Indian$ or Pakistan$ or Bangladesh$ or Bengal$).tw.
56. ((Asia$ or Pacific) adj4 American$).tw.
57. ((Asia$ or Pacific) adj4 Islander$).tw.
58. (Hispanic or Latino or Latin American$ or Puerto Ric$ or Mexican$).tw.
59. or/21-58
60. program evaluation/
61. program$.tw.
62. ((educat$ or behaviour$ or behavior$ or improv$) adj3 (intervention$ or activat$)).tw.
63. health care delivery/
64. patient attitude/
65. health promotion/
66. (health adj2 (promotion$ or knowledge or belief$)).tw.
67. health education/
68. (educat$ adj2 (information or material or leaflet)).tw.
69. attitude to health/
70. health behavior/
71. patient education/
72. persuasive communication/
73. questionnaires/
74. information processing/
75. health survey/
76. health care survey/
77. exp interview/
78. (questionnaire$ or survey$).tw.
79. (focus adj3 group$).tw.
80. reminder system/
81. remind$.tw.
82. telephone/
83. telephone interview/
84. telephone.tw.
85. phone call.tw.
86. health behavior/
87. behavior therapy/
88. behavior change/
89. (behavioral or behavior or behaviour or behavioural).tw.
90. (increas$ adj3 (attend$ or uptake)).tw.
91. (approachability or acceptability or availability or affordability or appropriateness).tw.
92. (ability adj2 (perceive or seek or reach or pay or engage)).tw.
93. vision test/
94. mass screening/
95. (vision adj3 (test$ or screen$ or assess$)).tw.
96. (eye$ adj3 (test$ or screen$ or assess$)).tw.
97. ((target$ or tailor$) adj3 intervention$).tw.
98. (cultural$ adj3 (sensitiv$ or appropriate)).tw.
99. or/60-98
100. 20 and 59 and 99
101. exp developing country/
102. 100 not 101
103. prevalence.ti.
104. (genetic or mutation$ or autosomal or variant$).ti.
105. optical coherence tomography.tw.
106. (Latin American and Caribbean Health Sciences).tw.
107. or/103-106
108. 102 not 107
109. exp case report/
110. (case$ adj3 (report$ or series)).tw.
111. or/109-110
112. 108 not 111
113. limit 112 to conference abstract status
114. 112 not 113

For peer review only - http://bmjopen.bmj.com/site/about/guidelines.xhtml

Global Health (Ovid)

1. eyes/
2. eye diseases/
3. vision/
4. vision disorders/
5. ((eye$ or ocular or vision) adj2 (care or health or service$)).tw.
6. visual acuity.tw.
7. (ophthalm$ or optomet$).tw.
8. (glaucoma$ or ocular hypertension or cataract$).tw.
9. ((diabet$ or proliferat$) adj3 retinopath$).tw.
10. (diabet$ adj3 (eye$ or vision or visual$ or sight$)).tw.
11. (retinopath$ adj3 (eye$ or vision or visual$ or sight$)).tw.
12. (dilated adj2 fundus).tw.
13. (retinal adj2 exam$).tw.
14. (myop$ or hyperop$ or hypermetrop$ or anisometrop$ or ammetrop$ or astigmati$ or presbyop$).tw.
15. (refractive adj1 error$).tw.
16. (spectacle or spectacles or glasses).tw.
17. (eyeglasses or eye glasses).tw.
18. or/1-17
19. ethnic groups/
20. ethnicity/
21. minorities/
22. ((ethnic$ or racial$ or cultur$) adj3 (group$ or minorit$ or population$ or diverse$ or origin$)).tw.
23. ((ethnic$ or racial$ or cultur$) adj3 (inequalit$ or inequit$ or disparit$ or equit$ or disadvantage$ or depriv$)).tw.
24. ((population$ or communit$) adj3 (divers$ or disadvantage$ or depriv$)).tw.
25. non-Indigenous.tw.
26. (visible adj1 minorit$).tw.
27. refugees/
28. immigrants/
29. immigration/
30. (migrant$ or immigrant$ or emigrant$ or refugee$ or expatriate$).tw.
31. asylum seeker$.tw.
32. urban population/
33. urban areas/
34. urban poor.tw.
35. geography.tw.
36. african americans/ or african-caribbeans/ or american indians/ or asians/ or black people/ or japanese americans/ or mexican-americans/ or pacific islanders/
37. (Afr$ adj2 American$).tw.
38. (Afr$ adj2 Caribbean$).tw.
39. (west adj2 (india$ or indies)).tw.
40. (American$ adj1 black).tw.
41. (Asian$ or Indian$ or Pakistan$ or Bangladesh$ or Bengal$).tw.
42. ((Asia$ or Pacific) adj4 American$).tw.
43. or/19-42
44. program evaluation/
45. programs/
46. program$.tw.
47. ((educat$ or behaviour$ or behavior$ or improv$) adj3 (intervention$ or activat$)).tw.
48. (primary health care or health services or community health or health policy or health care or public health or health programmes).sh.
49. health promotion/
50. (health adj2 (promotion$ or knowledge or belief$)).tw.
51. health education/
52. patient education/
53. (educat$ adj2 (information or material or leaflet)).tw.
54. attitude/
55. communication/
56. questionnaires/
57. discussion groups/
58. surveys/
59. (questionnaire$ or survey$).tw.
60. (focus adj3 group$).tw.
61. remind$.tw.
62. telephone/
63. telephone.tw.
64. phone call.tw.

For peer review only - http://bmjopen.bmj.com/site/about/guidelines.xhtml

65. behaviour/
66. (behavioral or behavior or behaviour or behavioural).tw.
67. (increas$ adj3 (attend$ or uptake)).tw.
68. (approachability or acceptability or availability or affordability or appropriateness).tw.
69. (ability adj2 (perceive or seek or reach or pay or engage)).tw.
70. screening/
71. (vision adj3 (test$ or screen$ or assess$)).tw.
72. (eye$ adj3 (test$ or screen$ or assess$)).tw.
73. ((target$ or tailor$) adj3 intervention$).tw.
74. (cultural$ adj3 (sensitiv$ or appropriate)).tw.
75. culture/
76. or/44-75
77. 18 and 43 and 76
78. exp developing countries/
79. 77 not 78
80. prevalence.ti.
81. (genetic or mutation$ or autosomal or variant$).ti.
82. optical coherence tomography.tw.
83. (Latin American and Caribbean Health Sciences).tw.
84. or/80-83
85. 79 not 84
86. exp case report/
87. (case$ adj3 (report$ or series)).tw.
88. 86 or 87
89. 85 not 88

For peer review only
